# Novel in vitro booster vaccination to rapidly generate antigen-specific human monoclonal antibodies

**DOI:** 10.1084/jem.20170633

**Published:** 2017-08-07

**Authors:** Irene Sanjuan Nandin, Carol Fong, Cecilia Deantonio, Juan A. Torreno-Pina, Simone Pecetta, Paula Maldonado, Francesca Gasparrini, Jose Ordovas-Montanes, Samuel W. Kazer, Svend Kjaer, Daryl W. Borley, Usha Nair, Julia A. Coleman, Daniel Lingwood, Alex K. Shalek, Eric Meffre, Pascal Poignard, Dennis R. Burton, Facundo D. Batista

**Affiliations:** 1 Lymphocyte Interaction Laboratory, Francis Crick Institute, London, England, UK; 2 Protein Purification and Structural Biology, Francis Crick Institute, London, England, UK; 3 Ragon Institute of Massachusetts General Hospital, MIT, and Harvard, Cambridge, MA; 4 Broad Institute of MIT and Harvard, Cambridge, MA; 5 Institute for Medical Engineering and Science, MIT, Cambridge, MA; 6 Department of Chemistry, MIT, Cambridge, MA; 7 Diagnostic and Molecular Development, hLAB Division, hVIVO PLC, Queen Mary BioEnterprises Innovation Centre, London, England, UK; 8 Division of Health Sciences and Technology, Harvard Medical School, Boston, MA; 9 Department of Immunobiology, Yale University School of Medicine, New Haven, CT; 10 International AIDS Vaccine Initiative Neutralizing Antibody Center and the Collaboration for AIDS Vaccine Discovery, The Scripps Research Institute, La Jolla, CA; 11 Department of Immunology and Microbial Science, The Scripps Research Institute, La Jolla, CA

## Abstract

Vaccines remain the most effective tool to prevent infectious diseases. Here, we introduce an in vitro booster vaccination approach that relies on antigen-dependent activation of human memory B cells in culture. This stimulation induces antigen-specific B cell proliferation, differentiation of B cells into plasma cells, and robust antibody secretion after a few days of culture. We validated this strategy using cells from healthy donors to retrieve human antibodies against tetanus toxoid and influenza hemagglutinin (HA) from H1N1 and newly emergent subtypes such as H5N1 and H7N9. Anti-HA antibodies were cross-reactive against multiple subtypes, and some showed neutralizing activity. Although these antibodies may have arisen as a result of previous influenza infection, we also obtained gp120-reactive antibodies from non–HIV-infected donors, indicating that we can generate antibodies without prior antigenic exposure. Overall, our novel approach can be used to rapidly produce therapeutic antibodies and has the potential to assess the immunogenicity of candidate antigens, which could be exploited in future vaccine development.

## Abstract (precis)

Sanjuan Nandin et al. describe an innovative approach based on antigen-dependent activation of human memory B cells in culture. It results in the rapid generation of human antibodies against infectious agents and offers the potential for therapeutic antibody production and vaccine development.

## Introduction

B lymphocytes (B cells) play a critical role in adaptive immunity, providing protection from pathogens through the production of specific antibodies. B cells recognize and respond to pathogen-derived antigens through surface B cell receptors (BCRs). The BCR has two interrelated functions in B cell activation. The first is to initiate signal cascades that result in the transcription of a variety of genes associated with B cell activation (Pierce and Liu, 2010). The second is to mediate antigen uptake and processing, leading to antigen presentation to T cells within the MHC class II context and full activation of the B cells (Lanzavecchia, 1985). Similarly, BCR-mediated antigen internalization has been shown to facilitate the presentation of lipid antigens in the context of CD1d, which can result in the recruitment of iNKT cell help (Barral et al., 2008; Leadbetter et al., 2008) or the transport of TLR agonists, resulting in TLR7 or TLR9 signaling (Marshak-Rothstein, 2006; Hou et al., 2011).

TLRs recognize structurally conserved sequences in pathogen-associated ligands, provide costimulation to immune cells, and are involved in promoting B cell responses and also in autoimmunity (Leadbetter et al., 2002; Pasare and Medzhitov, 2005; Christensen et al., 2006; DeFranco et al., 2012; Shlomchik and Weisel, 2012). In mice, it has long been known that, even in the absence of BCR signaling or T cell help, naive B cells can undergo proliferation and differentiation in response to TLR ligands such as LPS and CpG (Coutinho et al., 1974; Krieg, 2002; Eckl-Dorna and Batista, 2009). In human B cells, TLR signaling has been suggested to represent a third signal required for the polyclonal activation of naive B cells (Ruprecht and Lanzavecchia, 2006). Furthermore, TLR signaling has also been implicated in antibody responses in vivo, long-term B cell memory, and plasma cell differentiation (Bernasconi et al., 2002). Similarly, stimulation of B cells via TLR ligands has been associated with promotion of plasma cell differentiation (Rawlings et al., 2012). However, the precise signaling requirements that promote terminal B cell differentiation are a topic of intense investigation (Nutt et al., 2015).

In recent years, the potent immunostimulatory properties of CpG oligodeoxynucleotides (CpG-ODNs) have been exploited in the study of human antibody responses. It has been reported that CpG DNA can enhance the efficiency of EBV-immortalization of B cells (Traggiai et al., 2004; Yu et al., 2008b). Furthermore, the use of such EBV-transformed human B cells in fusions can increase hybridoma formation as much as 25-fold compared with untransformed PBMCs (Yu et al., 2008b). These strategies have not only led to the generation of neutralizing antibodies against the influenza strain responsible for the 1918 pandemic (Yu et al., 2008b), but have also been exploited to study antibody responses to many pathogens, including CMV (Macagno et al., 2010), influenza virus (Yu et al., 2008a; Corti et al., 2010), HIV (Buchacher et al., 1994), and dengue virus (Dejnirattisai et al., 2010; Smith et al., 2014).

Soluble oligonucleotides containing unmethylated CpG have, therefore, been used to expand human B cell populations in vitro from infected or vaccinated individuals. However, this strategy is laborious and time consuming, as extensive screening is needed to retrieve the comparatively rare antigen-specific B cells contained within this expanded B cell population. During the last decade, the direct cloning of Ig variable genes from single cells (Babcook et al., 1996; Wardemann et al., 2003) and, more recently, the next-generation sequencing of IgH variable genes have facilitated the isolation of antigen-specific B cells from the plasmablast or memory cell population in the peripheral blood approximately a week after infection or vaccination (Wrammert et al., 2008; Scheid et al., 2009; Smith et al., 2009; Zhu et al., 2013). One limitation in using some of these antigen-specific B cell isolation methods is imposed by the time frame of the humoral response to infection or immunization, which typically peaks 7 d postvaccination and returns to barely detectable levels of specific antibody by day 14 (Wrammert et al., 2008). The results of these in vivo studies underscore the need for fast and inexpensive in vitro systems that can recapitulate at least some features of the human immune response.

Antigen-specific B cell activation is a key step in the initiation of immune responses. The in vitro activation of B cells in an antigen-dependent manner is difficult to achieve, because the wide haplotype variation of MHCIIs necessitates the use of unique T cells specific to a particular MHCII to activate B cells in vitro (Fauci et al., 1976; Borrebaeck et al., 1988). To overcome these limitations, we developed a novel, in vitro strategy to stimulate human B cells with streptavidin nanoparticles conjugated to both GpG and antigen (Eckl-Dorna and Batista, 2009). B cells producing antigen-specific antibodies were identified, quantified, and characterized to determine the antibody repertoire. We validated the utility of this approach using human B cells from healthy individuals against a wide range of infectious targets including tetanus toxoid (TT), hemagglutinin (HA) from several subtypes of influenza A, and the HIV envelope protein gp120. We also determined the physiological effectiveness of the antibodies by measuring their antigen-binding affinities and neutralizing abilities. Additionally, we demonstrated that this method allows selective, in vitro recall of human B cells, overcoming the difficulties of either isolating the infrequent antigen-specific memory B cells present in the peripheral blood of healthy subjects under normal circumstances or the requirement for vaccination or infection and the use of plasmablasts as a source of antigen-specific cells. This is a novel and valuable approach not only to rapidly generate therapeutic antibodies, but also to accelerate immunogen design, testing, and early characterization of human vaccines, obviating the need for laborious, time-consuming, and expensive clinical trials.

## Results

### In vitro stimulation of memory B cells by particulate CpG in a BCR-dependent manner

Previous studies have demonstrated that the in vitro stimulation of human B cells by TLRs is an efficient way of inducing the activation and proliferation of these cells, irrespective of BCR specificity (Bernasconi et al., 2003; Pinna et al., 2009). These findings have been exploited as an efficient way of retrieving human monoclonal antibodies in vitro (Traggiai et al., 2004). We hypothesized that the delivery of a TLR ligand via BCR-mediated internalization might represent a way of exclusively activating B cells with a particular BCR specificity. To determine the best stimulatory conditions for such activation, we first took advantage of an anti–κ-chain antibody to target CpG to the BCR, because 60% of circulating human B cells bear this BCR chain; the rest bear a λ chain. We coated streptavidin polystyrene nanoparticles with a mixture of biotinylated anti-κ antibody and the TLR ligand CpG, as we have previously described in mice (Eckl-Dorna and Batista, 2009).

**Figure 1.**
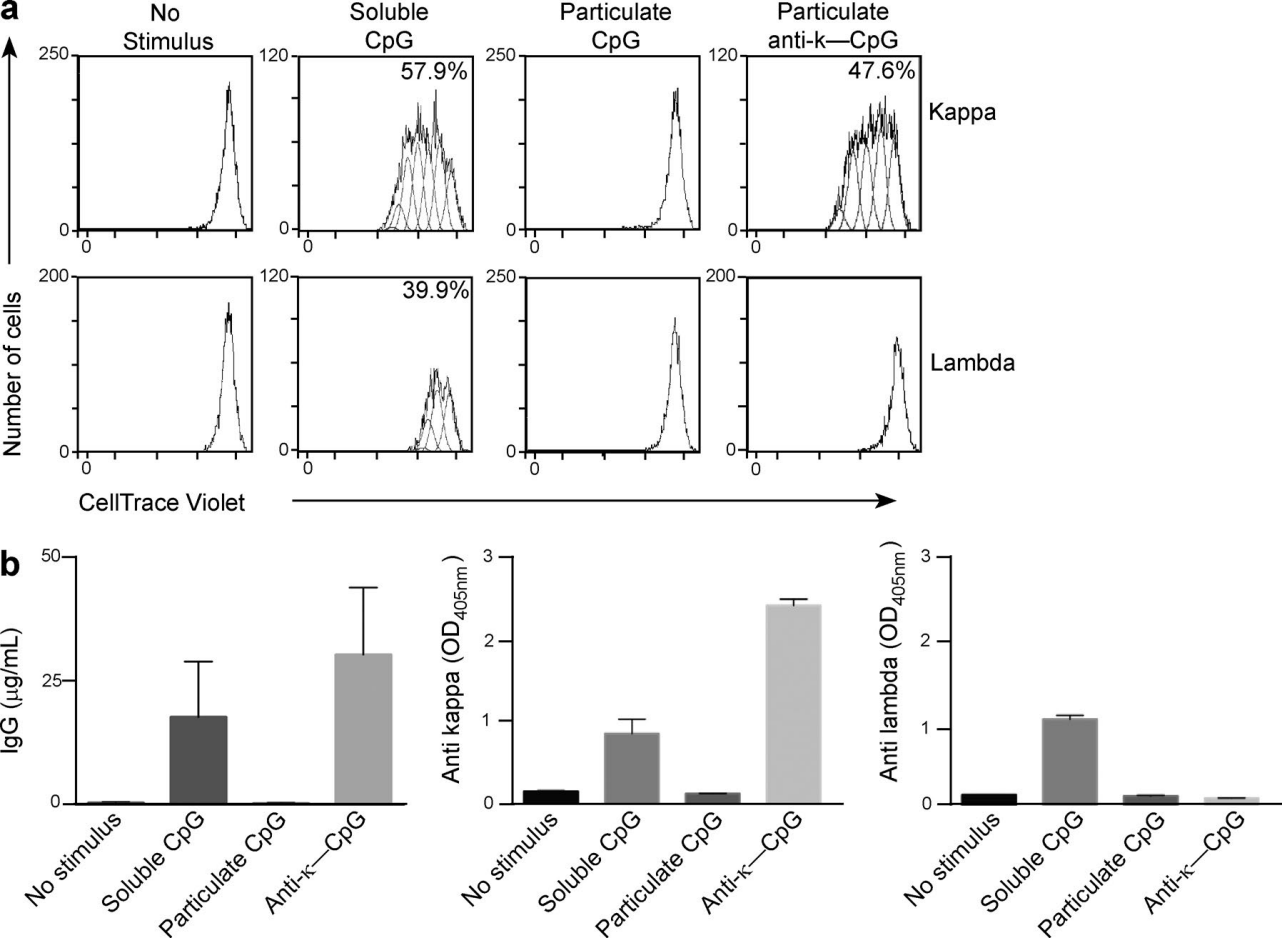
**In vitro stimulation with particulate anti–BCR-CpG enhances specific B cell proliferation and antibody secretion.** (a) CellTrace Violet–labeled cells were either not stimulated or stimulated for 6 d with soluble CpG, particulate CpG, or particulate anti–κ-CpG. Flow cytometry was used to measure proliferation, by CellTrace Violet dilution, of memory B cells bearing BCRs containing either the κ or λ chain. Numbers in the boxes indicate the percentage of proliferating cells. One representative result of three independent experiments is shown. (b) Memory B cells were cultured in the presence of different stimulatory conditions or no stimulation. The concentration of IgG antibodies secreted in the culture supernatant and the presence of κ- or λ-chain–bearing antibodies was determined by ELISA. Left, mean ± SD IgG concentrations from eight different donors. Middle and right, ELISA measurements of κ- and λ-chain Igs, respectively. Results represent the mean OD_405_ values ± SD of two replicates; one representative result of eight independent experiments is shown.

To determine whether we could use these nanoparticles in vitro to selectively activate only those memory B cells expressing a κ-chain BCR, CellTrace Violet–labeled human memory B cells obtained from healthy donors were cultured in the presence of a cytokine cocktail with nanoparticles coated with either anti-κ and CpG (anti–κ-CpG) or CpG alone; soluble CpG; or no stimulant as a control. 6 d after stimulation, flow cytometry was used to measure B cell proliferation by the dilution of the CellTrace Violet. As expected, soluble CpG induced a robust proliferation of B cells irrespective of whether they expressed κ- or λ-BCR (57.9% vs. 39.9%, respectively; Fig. 1 a). In contrast, CpG that had been rendered particulate did not trigger any proliferation unless anti-κ was also present. Particulate anti–κ-CpG could stimulate only B cells with a κ-BCR, leading to 47.6% proliferation (Fig. 1 a). These results show that when internalized via the BCR, particulate CpG can be used to selectively activate memory B cells bearing a particular BCR in vitro.

To investigate whether this type of stimulation could also lead to antibody secretion, we measured the total IgG production in the culture supernatants by ELISA 6 d poststimulation. Stimulation with particulate CpG did not induce any detectable antibody in the culture supernatant. In contrast, we detected an increase in IgG secretion when the B cells were stimulated with either soluble CpG or anti–κ-CpG (Fig. 1 b, left). As expected, this increase in IgG levels corresponded with a similar increase in IgG antibodies bearing the κ-light chain (κ-Ig; Fig. 1 b, middle). Importantly, no λ antibodies (λ-Ig) could be detected in the supernatant of B cells stimulated with anti–κ-CpG, indicating that the κ-Igs we detected were the result of specific κ-BCR targeting (Fig. 1 b, right). Therefore our results indicate that the in vitro targeting of particulate CpG via BCR triggers selective antibody production.

**Figure 2.**
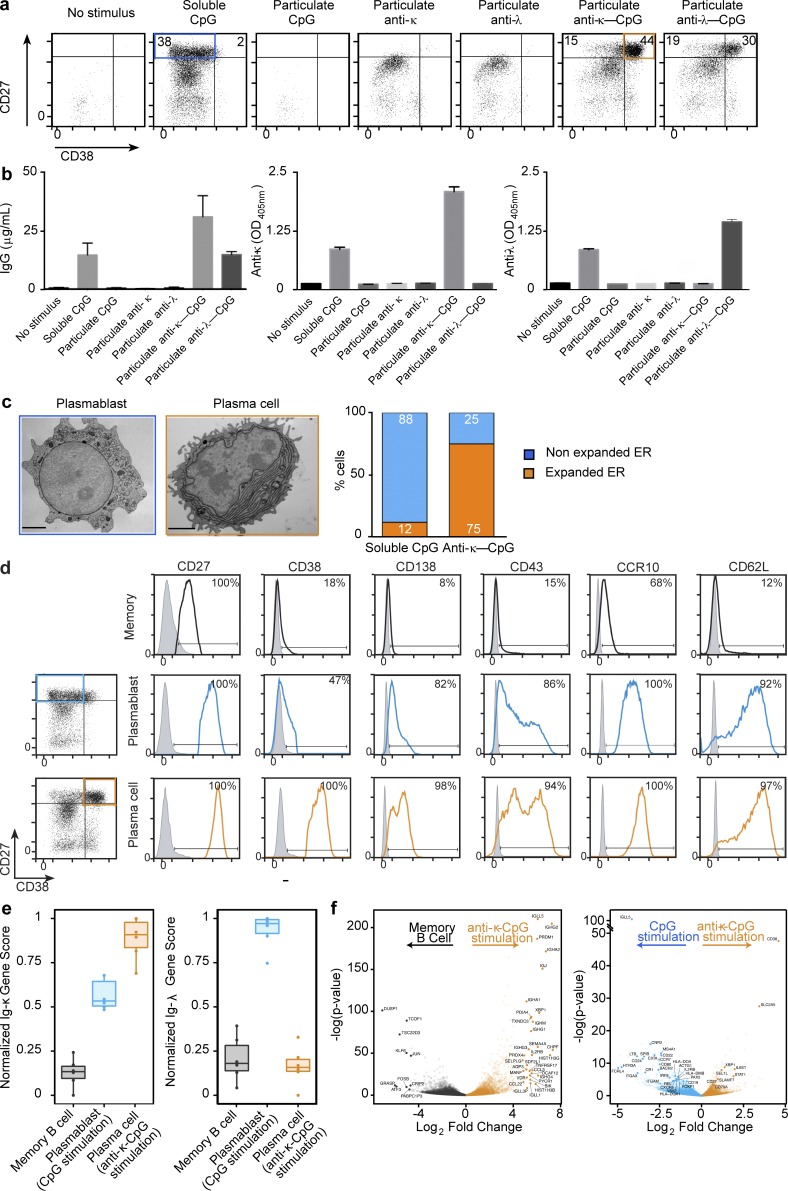
**Uptake of particulate anti–BCR-CpG is essential for promoting CD27^hi^/CD38^hi^ plasma cell differentiation.** (a) Flow cytometry profiles of CellTrace Violet–labeled memory B cells unstimulated or cultured for 6 d in the presence of soluble GpG, particulate CpG, particulate anti-BCR, or particulate anti–BCR-CpG. Numbers in the plots indicate the percentage of plasmablasts (blue) and plasma cells (orange) based on the expression of CD27/CD38 in proliferating cells. One representative result of six independent experiments is shown. (b) The concentration of IgG antibodies secreted in the culture supernatant and the presence of κ- or λ-chain antibodies was determined by ELISA. Left, mean ± SD IgG concentrations from three different donors. Middle and right, results of ELISA assays to detect κ- and λ-Igs, respectively. Results represent the mean OD_405_ values ± SD of two replicates; one representative result of three independent experiments is shown. (c) Representative TEM images of CD27^hi^/CD38^int^ plasmablasts and CD27^hi^/CD38^hi^ plasma cells of six independent experiments. Bars, 2 µm. Percentage of cells with expanded and nonexpanded ER is shown in the chart to the right (*n* = 100). (d) Comparison of the surface phenotype of the CD27^hi^/CD38^int^ (blue) and CD27^hi^/CD38^hi^ (orange) cell populations after particulate anti–κ-CpG stimulation. As a control, the phenotype of the memory B cells before stimulation is also shown. Flow cytometry data from one representative experiment of four independent experiments is shown. In each plot, gray traces represent the corresponding isotype control. Numbers in panels indicate the percentage of cells positive for the indicated markers. (e) Specific activation of memory B cells by anti–κ-CpG leads to the acquisition of a plasma cell phenotype revealed by RNA-seq analysis, performed on mRNA isolated from memory B cells that were unstimulated or stimulated with CpG or anti–κ-CpG. Transcriptional profiles of each B cell population were scored against Ig-κ and Ig-λ variable and constant region genes. Box and whiskers represent the upper and lower quartile of scores and 1.5 × the inner quartile range, respectively. (f) Significance plotted against log2 fold change for differentially expressed genes as determined by DESeq2 analysis between B cells stimulated with anti–κ-CpG and unstimulated B cells and between B cells stimulated with anti–κ-CpG and CpG. For this experiment, data are derived from PBMCs obtained from six independent donors.

Interestingly, we noted that although soluble CpG stimulation induced B cell proliferation, the amount of κ-Igs secreted in these cultures was lower than that produced in response to anti–κ-CpG. We wondered whether this could be caused by B cells reaching different stages of plasma cell differentiation in each of these conditions. To investigate this, we used flow cytometry to monitor the differentiation of proliferating cells by the expression of the surface molecules CD27 and CD38, which are up-regulated during plasma cell differentiation. When stimulated with soluble CpG, 38% of the cells were CD27^hi^/CD38^int^, a characteristic phenotype that has been described for plasmablasts (Fig. 2 a; Fink, 2012). Stimulation with particulate anti–κ-CpG or anti–λ-CpG (but not with particulate anti-κ or anti-λ) led to the production of CD27^hi^/CD38^hi^ cells (44% for anti–κ-CpG and 30% for anti–λ-CpG), characteristic of a plasma cell phenotype (Fig. 2 a). In line with the plasma cell differentiation stage, we observed an increase in IgG secretion after stimulation with soluble CpG and particulate anti–κ-CpG or anti–λ-CpG and higher levels of IgG antibodies bearing κ-light or λ chain when the cells were stimulated with particulate anti–κ-CpG or anti–λ-CpG, respectively (Fig. 2 b). Comparable results were obtained using memory B cells isolated from fresh and cryopreserved PBMCs (Fig. S1). The generation of plasmablasts and plasma cells in the different cultures was further confirmed and quantified by transmission electron microscopy (TEM). Unlike plasmablasts, plasma cells exhibit an expanded ER, a feature typically associated with large-scale antibody production. We found that the majority (88%) of the CD27^hi^/CD38^int^ cells showed typical plasmablast morphology, with no changes in the ER. In contrast, 75% of the CD27^hi^/CD38^hi^ cells possessed expanded ER, indicating that the CD27^hi^/CD38^hi^ cells were indeed plasma cells (Fig. 2 c).

To further characterize the CD27^hi^/CD38^int^ population generated after soluble CpG stimulation and the CD27^hi^/CD38^hi^ population obtained after anti–κ-CpG stimulation, we analyzed the expression of specific cell surface markers in these populations by flow cytometry. The CD27^hi^/CD38^hi^ population displays a plasma cell phenotype characterized by higher levels of CD138 and CD43 compared with the CD27^hi^/CD38^int^ population (Fig. 2 d). Consistent with plasma cell differentiation, we also observed a lower expression of CD19, CD20, IgD, HLA-DR, CD80, and CD86 (Fig. S2 a). With regard to the cell surface expression of homing receptors, CD27^hi^/CD38^hi^ cells are characterized by an increase in the mucosal or epidermal chemokine receptor, CCR10, and the L-selectin, CD62L (Fig. 2 d), and a reduction in the chemokine receptors CXCR4 and CXCR5 compared with the CD27^hi^/CD38^int^ population (Fig. S2 a).

We examined the gene expression profiles of the CD27^hi^/CD38^hi^ versus CD27^hi^/CD38^int^ cell populations by quantitative real-time PCR. As expected, the two cell populations substantially down-regulate key transcription factors such as PAX5 and BCL6, which are required for naive B cell maintenance. Similarly, when compared with the CD27^hi^/CD38^int^ population, CD27^hi^/CD38^hi^ cells expressed higher levels of transcription factor (TF) genes such as XBP1, IRF4, and PRMD1, which are required for plasma cell differentiation (Fig. S2 b). We also analyzed the early molecular signaling pathways triggered after the activation of memory B cells with the different stimuli, particulate anti–κ-CpG, particulate anti-κ, and soluble CpG, by evaluating the activation status of a range of intracellular mediators such as pAKT, pERK, pSTAT3, and p-p38 in response to these stimuli. We observed that in agreement with a previously published study (Eckl-Dorna and Batista, 2009), compared with memory B cell stimulation with soluble CpG or particulate anti-κ, particulate anti–κ-CpG stimulation resulted in more sustained phosphorylation of p38 (Fig. S2 c). However, stimulation with particulate anti–κ-CpG did not induce a synergistic effect in the phosphorylation of AKT, ERK, or STAT3 (Fig. S2 c).

To support our findings from flow cytometry, TEM, and molecular signaling characterization that anti–κ-CpG stimulation preferentially leads to the generation of κ-IgG–secreting plasma cells, we performed RNA-seq analysis on memory B cells (day 0) and FACS-sorted B cells stimulated with CpG and anti–κ-CpG (day 6) from five donors. An analysis of the transcriptional activity of all Ig-κ and Ig-λ variable and constant region genes revealed that anti–κ-CpG stimulation resulted in the specific enhanced expression of κ-chain genes in CD27^hi^/CD38^hi^ cells, with minimal bystander activation of λ-chain B cells (Fig. 2 e). Furthermore differential expression across the entire transcriptome confirms the up-regulation of genes involved in antibody production and plasma cell markers in B cells stimulated with anti–κ-CpG in comparison to memory B cells and CpG-stimulated B cells (plasmablasts), respectively (Fig. 2 f). Collectively, our experimental results suggest that the targeting of particulate CpG via the BCR can work as a selective method for stimulating human memory B cells to proliferate and differentiate into CD27^hi^/CD38^hi^ plasma cells, leading to antibody production.

### Enrichment of TT-specific antibodies in cultures of human memory B cells

**Figure 3.**
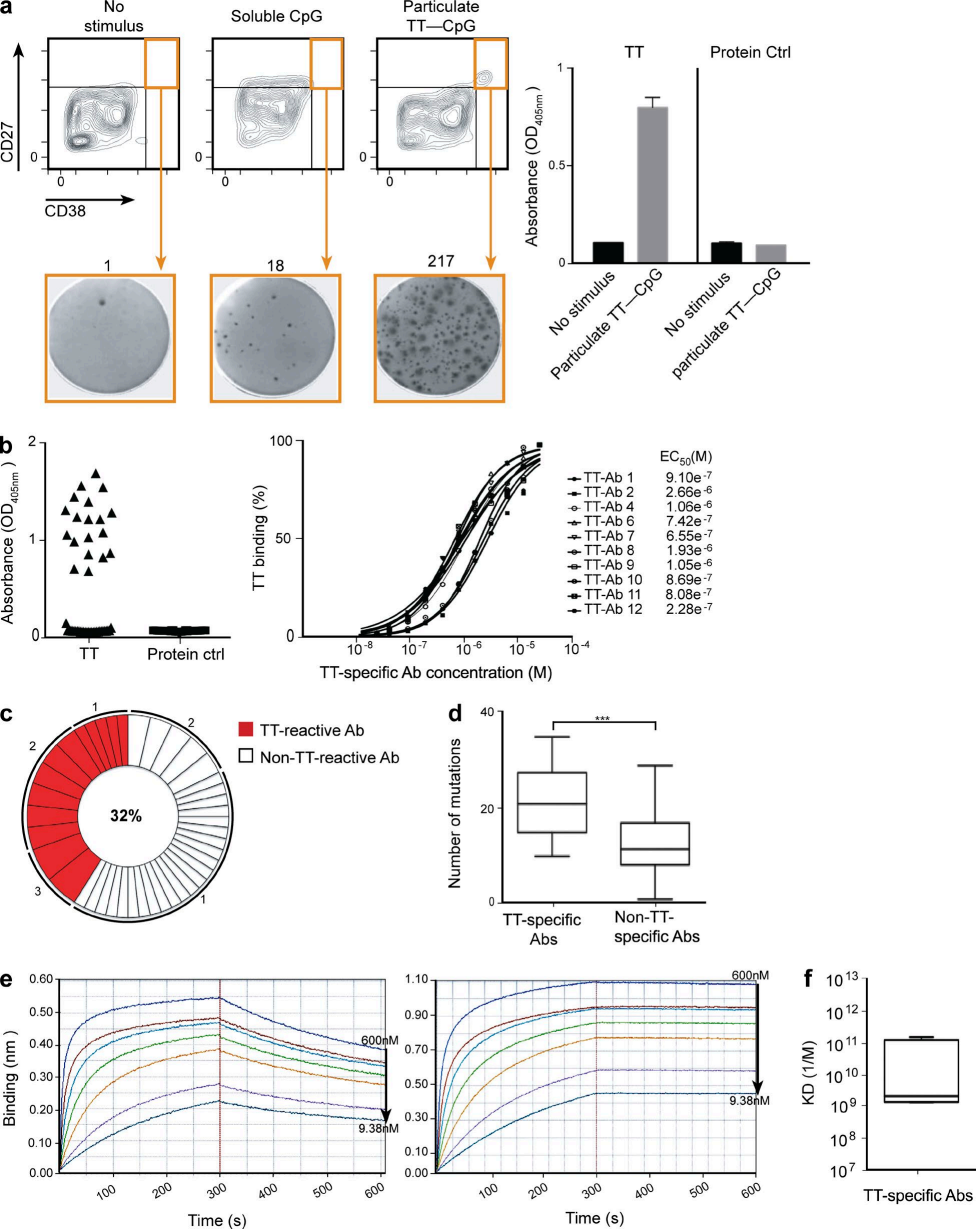
**Generation of human TT-specific antibodies from sorted plasma cells.** (a) Representative FACS profile and gating strategy for sorting plasma cells (orange box) after no stimulation or stimulation with soluble CpG or particulate TT-CpG. Enrichment of TT-specific plasma cells after sorting was determined by ELISPOT and ELISA. One representative ELISPOT of three shows IgG-producing plasma cells that are reactive against TT. The numbers above the ELISPOT panels represent the spot count for that well. Culture supernatants from sorted plasma cells were tested by ELISA (right) for the presence of antibodies specific for TT or to an unrelated protein control. Data represent the mean OD_405_ values ± SD from two replicates. Data from one representative experiment of three independent repeats is shown. (b) The specificity of the recombinant antibodies generated from 56 single-sorted plasma cells was tested by ELISA. Cognate IgH and IgL gene pairs were transiently transfected into HEK293T cells, and the culture supernatant was tested for the presence of antibodies specific for TT or an unrelated protein control (left); the mean IgG antibody concentration was 1 µg/ml. The cutoff value for a positive signal was set as more than twofold above background. The EC_50_ values of the transfected TT-specific antibodies were extracted from the fitting of the raw data to a sigmoidal multiparameter curve (right). (c) Pie chart outlines the frequency of the TT-specific clones. The percentage of TT-positive antibodies is denoted in the center of the pie chart. The numbers outside the pie chart indicate the number of antibody allele sequences with identical IgH and IgL chain rearrangements. (d) Comparison of the absolute numbers of somatic mutations in the VH genes encoding 13 TT-specific and 24 non–TT-specific antibodies. Boxes represent the percentile range (25–75%), the horizontal bar indicates the median, and whiskers extend to the highest and lowest data points. Two-tailed P values were calculated with an unpaired *t* test (***, P = 0.0005). (e) Representative Octet measurements of the kinetics of TT-specific antibodies (left, K_d_ = 6.64 × 10^−9^; right, K_d_ = 1.85 × 10^−11^). Concentrations of the antibodies represent serial twofold dilutions. (f) K_d_ of TT-specific antibodies. Boxes represent the percentile range (25–75%), the horizontal bar indicates the median KD value (1.6 nM) of six TT-specific antibodies, and whiskers extend to the highest and lowest data points.

Having demonstrated the potential of particulate anti–κ-CpG in triggering plasma cell differentiation and antibody secretion, we hypothesized that we could extend these findings and use this protocol to specifically activate B cells with infectious antigens in vitro. Accordingly, we first chose TT as an antigen. The percentage of TT-specific memory cells in circulating blood of immunized individuals has been estimated to be ∼0.04% (Giesecke et al., 2014), which is three orders of magnitude lower than the 60% of B cells bearing a κ-chain BCR. For this experiment, memory B cells were either unstimulated or stimulated with soluble CpG or particulate TT-CpG. It is important to note that memory B cells were purified from healthy donors who had not been recently vaccinated against tetanus or otherwise restimulated. Plasma cell differentiation was assessed by flow cytometry as described earlier. Interestingly, a distinct population of CD27^hi^/CD38^hi^ plasma cells was observed after stimulation with TT-CpG (Fig. 3 a, top row, orange quadrants). This plasma cell population is likely to be the result of BCR-specific recognition of particulate TT-CpG, as it was completely absent in the nonstimulated control or when B cells from the same donor were stimulated with soluble CpG alone (Fig. 3 a, top row, orange quadrants).

To establish that this CD27^hi^/CD38^hi^ population does indeed contain TT-specific antibody-secreting cells, and to get an estimate of their frequency, we sorted this population by FACS and performed TT-specific enzyme-linked immunospot (ELISPOT) assays. As shown in Fig. 3 a (lower row), 217 of the 2,500 plasma cells sorted after memory B cell stimulation with particulate TT-CpG were TT specific. In contrast, there was a 10-fold decrease in the number of plasma cells (*n* = 18/2,500) present after memory B cells were stimulated with soluble CpG. To test the specificity of the antibodies produced by bulk-sorted plasma cells after particulate TT-CpG stimulation, we performed ELISA using TT as a coating antigen (Fig. 3 a, right). We found that only antibodies produced after particulate TT-CpG stimulation were TT specific, and no signal was produced in the absence of stimulus. Moreover, the ELISA results showed that these antibodies were TT specific, and they did not exhibit any cross-reactivity to an irrelevant protein control (Fig. 3 a, right).

To unambiguously establish the number of TT-specific plasma cells present in these cultures, we performed single-cell sorting of CD27^hi^/CD38^hi^ plasma cells by FACS. For each cell, we amplified the corresponding heavy and light chains by nested PCR and cloned them into expression vectors. To produce recombinant monoclonal human antibodies of the same specificity in vitro, we transiently expressed 56 antibodies in HEK-293 cells. The culture supernatants were harvested after 3 d, quantified for expressed IgG levels, and screened for antibody specificity by ELISA (Fig. 3b, left). The levels of antibody production by individual transfections ranged from 0.5 to 3 µg/ml, with a mean concentration of 1 µg/ml. Furthermore, half-maximal binding (EC_50_) of the TT-specific antibodies was also determined and found to be in the micromolar range, reinforcing the TT specificity of these antibodies (Fig. 3 b, right).

The ELISA results and sequence analysis of the VH segments revealed 23 TT-specific IgGs of the 56 expressed antibodies (32%), with 13 unique sequences, as some of them were represented two or three times (Fig. 3 c and Table S1). Notably, this represents a >1,000-fold enrichment compared with the expected 0.04% circulating TT-specific memory B cells mentioned previously after only 6 d of in vitro stimulation (Giesecke et al., 2014). Moreover, the multiple encounter of the same antibody sequence is indicative of the in vitro proliferation of a single B cell after stimulation. These antibodies also showed an extensive diversity in V(D)J gene usage, with no dominant preferences for particular genes, and a broad distribution of heavy chain complementarity-determining region 3 (CDR3) lengths (Table S1). Some of the recovered VH segments also show sequence similarities to anti-TT VH gene sequences previously isolated from vaccinated individuals (de Kruif et al., 2009; DeKosky et al., 2013; unpublished data). Interestingly, we also found two antibodies with identical VH and JH gene usage and CDR3 junctions, but carrying single point mutations (TT-Ab3 and TT-Ab4; Table S1; and Fig. S3 a). These antibodies, although sharing identical heavy (VH1-69/D4-17/JH4) and light (VK-39/JK4) chain gene usage, differed in the number of mutations in their sequences, indicating that they are clonally related (Table S1; and Fig. S3 a). This is consistent with antibodies produced by individual B cells that most likely originated in vivo via somatic hypermutation but subsequently proliferated in vitro. The identification of 13 unique B cell clones strongly suggests that our in vitro booster vaccination is able to sample a wide repertoire of TT-specific B cells, in particular when taking into consideration that only single 56 activated B cells were used for single-cell sequence analysis.

To assess the possible contribution of somatic hypermutation in rearranged VH gene segments, the sequences from this region were compared with their respective germline IGHV genes. This analysis suggests the presence of mutations within framework regions (FWRs) 1–3 and CDR1/2 (Fig. S3 b). VH gene segments showed a higher number of mutations in TT-specific antibodies compared with sequences of unknown specificity (P = 0.005). The number of mutations ranged between 10 and 35 in TT-specific antibodies (21.77 ± 2.17) and between 1 and 29 in those of unknown specificity (12.75 ± 1.26; Fig. 3 d). An analysis of replacement (R) and silent (S) mutations in the TT-specific antibodies showed that there were more replacements than silent mutations in both the CDRs and the FRWs, although the CDRs displayed a higher overall R/S ratio (Fig. S3 b). Collectively, there was a higher number of mutations seen in the genes encoding TT-specific antibodies compared with those encoding non–TT-specific cloned antibodies, and a higher frequency of replacement mutations compared with silent mutations, especially in the CDRs, both of which suggest antigen-driven selection. Another possibility, however, is that particulate TT-CpG may have preferentially induced the differentiation or proliferation of a proportion of TT-specific memory cells with higher affinity or mutation.

Furthermore, we measured the affinity of the recombinant TT-specific antibodies by biosensor binding analysis (Fig. 3, e and f; and Table S2). The equilibrium dissociation constant (K_d_) values obtained revealed picomolar to nanomolar affinities (Fig. 3 e), with a median K_d_ of 1.6 nM (Fig. 3 f), indicating high-specificity binding for the antigen. Thus, despite the low numbers of TT-specific memory B cells in circulation in healthy individuals, we were able to specifically induce plasma cell activation and differentiation in vitro after only 6 d, allowing the recovery of several antibodies with high affinity for this antigen.

### Generation and characterization of antibodies to influenza A subtypes

**Figure 4.**
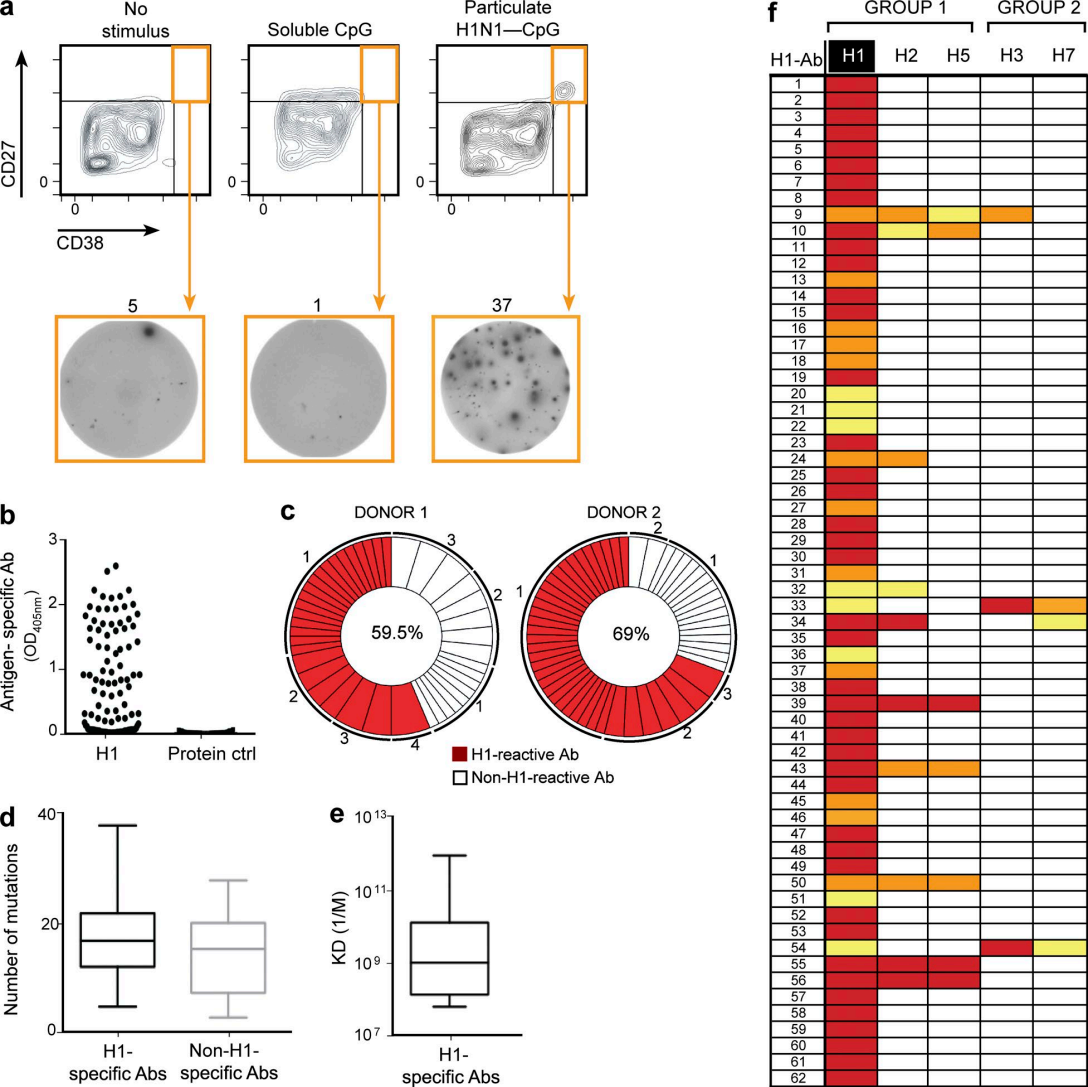
**Generation of human H1-specific antibodies from single-sorted plasma cells.** (a) Representative FACS profile and gating strategy for sorted plasma cells (orange box) after stimulation with soluble CpG, particulate H1-CpG, or no stimulation. Enrichment of FACS-sorted H1-specific plasma cells detected by ELISPOT. Numbers above the ELISPOT panels represent the spot counts for each well. Data from one representative experiment of three is shown. (b) Specificity of the recombinant antibodies generated from sorted plasma cells from two donors was tested by ELISA. After transient transfection of HEK293T cells with plasmids expressing cognate pairs of IgH and IgL genes, the culture supernatant was tested for the presence of antibodies specific for H1 or an irrelevant protein control; the recombinant IgG antibody concentration ranged from 1 to 3 µg/ml. (c) The frequency of the H1-specific clones is depicted by the pie chart. The percentage of H1-positive antibodies retrieved from each donor is indicated in the center of the pie chart. The numbers outside the pie chart indicate the number of antibody allele sequences with identical IgH and IgL chain rearrangements. (d) Comparison of the absolute numbers of somatic mutations in the VH genes encoding 56 H1-specific and 34 non–H1-specific antibodies. Boxes represent the percentile range (25–75%), the horizontal bar indicates the median, and whiskers extend to the highest and lowest data points. Two-tailed P values were calculated with an unpaired *t* test (P = 0.0870; NS). (e) Affinity (K_d_ 1/M) of the H1-specific antibodies. Boxes represent the percentile range (25–75%), the horizontal bar indicates the median K_d_ value (7.34 nM) of 14 H1-specific antibodies, and whiskers extend to the highest and lowest data points. (f) ELISA test to detect cross-reactivity of antibodies derived after H1 stimulation. Strong binding is coded in red (>2.0 OD_405_), intermediate in orange (1.0–2.0 OD_405_), and weak in yellow (0.3–1.0 OD_405_).

Having established the efficacy of our in vitro stimulation system using a protein that is a component of the standard vaccine program in infancy and childhood, we wanted to determine whether we could use it to generate antibodies against influenza A virus, a more complex natural pathogen. H1N1 is the most common influenza virus subtype infecting humans, so it is likely to have been encountered by many adults. To determine whether we could activate H1N1-specific B cells from individuals who had not been recently infected or vaccinated against influenza, we stimulated memory B cells from two healthy donors with particulate H1N1 HA (H1)-CpG or soluble CpG and BSA-CpG as controls. Similar to our observations using particulate TT-CpG, a defined CD27^hi^/CD38^hi^ plasma cell population was detected only when memory B cells were stimulated with particulate H1-CpG (Fig. 4 a, upper row, orange quadrants). This CD27^hi^/CD38^hi^ population contained a higher frequency (*n* = 37/10,000) of cells secreting anti-H1 antibody compared with the unstimulated (*n* = 5/10,000) or soluble CpG–stimulated (*n* = 1/10,000) memory B cells, as shown by the results of an H1-specific ELISPOT assay (Fig. 4 a, lower row).

We performed single-cell sorting of CD27^hi^/CD38^hi^ plasma cells obtained after particulate H1-CpG challenge. Next, we PCR amplified, cloned, and expressed 114 recombinant antibody genes from individual plasma cells from two donors in HEK293 cells. ELISA assays show that 65 of these antibodies, more than 50%, were specific for H1 (Fig. 4 b), indicating a high level of enrichment after only 6 d of in vitro stimulation. VH sequence alignment revealed 62 antibodies with unique sequences from the two donors (Figs. 4 c and S3). This analysis also revealed two clones that, although having identical gene usage in their VH and VL regions, exhibited different numbers of point mutations in their VH regions (Figs. S4 a). Interestingly our antibodies H1-Ab1 and H1-Ab2 show strong convergence, restriction, or selection to Ab 31.b.09, which has been recently isolated as a broadly neutralizing antibody capable of neutralizing H1, H5, H3, and H7 influenza subtypes. Additionally, we found that the same is true for H1-Ab3 antibody, isolated in this study, and Ab 01.k.01, which is capable of neutralizing H1, H5, and H3 (Joyce et al., 2016).

Analysis of somatic hypermutations in the rearranged VH gene segments of all the antibodies obtained showed that the genes encoding anti–H1-positive antibodies contain more mutations (17.61 ± 0.99) than the genes encoding anti–H1-negative antibodies (14.88 ± 1.20; Fig. 4 d) These mutations were mostly concentrated in the CDRs and displayed a higher R/S ratio compared with other regions, indicative of a positive antigen-driven selection (Fig. S4 b). Additionally, we determined the affinity of antigen-specific monoclonal antibodies for H1 by measuring the K_d_ via surface plasmon resonance. The resulting K_d_ values were in the nano- to picomolar range (median, 7.34 nM; Fig. 4 e and Table S4), indicating high-specificity binding for the antigen.

Antibody cross-reactivity among various influenza virus strains has been detected in several studies after infection and immunization with influenza vaccines (Corti et al., 2011; Ekiert et al., 2011). To check for reactivity against other influenza A subtypes, we screened the anti-H1 antibodies against HA from representative influenza subtypes from each of the two phylogenetically distinct groups: H1, H2N2 hemagglutinin (H2), and H5N1 hemagglutinin (H5) from group 1 and H3N2 hemagglutinin (H3) and H7N9 hemagglutinin (H7) from group 2. The majority of the antibodies reacted exclusively with antigenic H1; however, 12 antibodies showed cross-reactivity against at least one subtype (Fig. 4 f).

**Figure 5.**
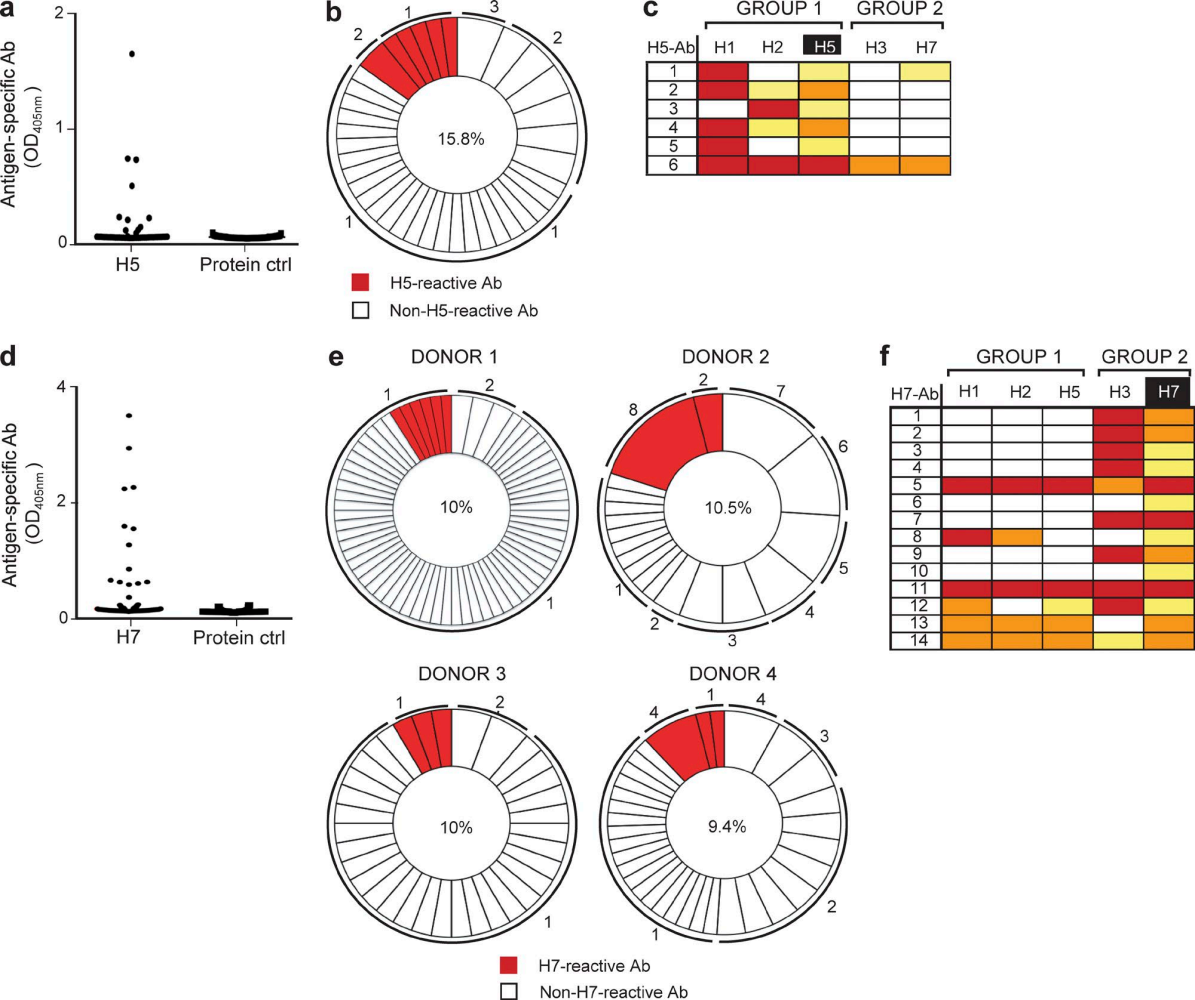
**Generation of human H5- and H7-specific antibodies from single sorted plasma cells.** (a) The specificity of H5-specific recombinant antibodies generated from sorted plasma cells from one donor was tested by ELISA. Cognate pairs of IgH and IgL genes were introduced into HEK293T cells by transient cotransfection. Culture supernatants were tested by ELISA for the presence of antibodies specific for H5 or an irrelevant protein control; the mean IgG antibody concentration was 1 µg/ml. Cutoff value for a positive signal is taken as more than twofold above background. (b) Pie chart outlining the frequency of the H5-specific clones. The number in the center indicates the percentage of H5-positive antibodies. The numbers outside the pie chart indicate the number of antibody gene sequences with identical IgH and IgL chain rearrangements. (c) ELISA test to detect cross-reactivity of antibodies derived after H5 stimulation. Strong binding is coded in red (>2.0 OD_405_), intermediate in orange (1.0–2.0 OD_405_), and weak in yellow (0.3–1.0 OD_405_). (d) Specificity of the H7 recombinant antibodies retrieved from four donors. Culture supernatants from transfected HEK293T cells, with a mean IgG concentration of 1 µg/ml, were tested by ELISA for the presence of antibodies specific for H7 or to an irrelevant protein control. The cutoff considered for positive results was more than twofold background. (e) Frequency of H7-specific clones. The number in the center of the pie chart indicates the percentage of H7-positive antibodies. The numbers outside the pie chart indicate the number of antibody alleles with identical IgH and IgL chain rearrangements. (f) ELISA analysis to detect cross-reactivity of antibodies derived after H7 stimulation. Strong binding is coded in red (>2.0 OD_405_), intermediate in orange (1.0–2.0 OD_405_), and weak in yellow (0.3–1.0 OD_405_).

Next, we wanted to address whether we could also generate antigen-specific antibodies using the rare influenza subtypes H5 and H7 that would also cross react with our panel of different influenza subtypes. To this end, we stimulated memory B cells of healthy donors with the uncommon H5N1 influenza HA. After 6 d of stimulation with particulate H5-CpG or BSA-CpG control, we sorted single CD27^hi^/CD38^hi^ plasma cells and cloned and expressed genes encoding 47 recombinant antibodies, seven (15%) of which were specific for H5 (Fig. 5 a). Sequence alignment analysis of the expressed alleles revealed six different H5-specific antibody gene sequences (Fig. 5 b and Table S5). The analysis of somatic hypermutations revealed that the alleles encoding H5-positive antibodies contained more mutations (24.40 ± 4.27) than those encoding H5-negative antibodies (12.88 ± 1.01; Fig. S4 c), indicating in vivo antigen-driven selection. Similar to our observations with H1, H5-specific antibody alleles displayed a higher R/S ratio in the CDRs than in FRW regions (Fig. S4 d). ELISA assays of these H5-specific antibodies against other influenza subtypes revealed a high degree of cross-reactivity with other HA proteins (Fig. 5 c). Indeed, H5-Ab6 was panreactive across all the subtypes tested, H5-Ab1 was cross-reactive with H1 and H7, and the remaining four were cross-reactive only with the group 1 strains, H1 and H2 (Fig. 5 c).

Similarly, using H7-HA as the antigen, we stimulated B cells from four different donors, single-cell sorted plasma cells, and sequenced their VH and VL regions. This analysis revealed 206 different antibodies, 14 of which proved to be positive for H7 (Fig. 5, d and e; and Table S6). Analysis of somatic hypermutation showed that the genes encoding H7-specific antibodies had more somatic mutations than those of unknown specificity. The number of mutations ranged from 11 to 35 in H7-specific cells (20.14 ± 1.95) and from 0 to 28 mutations in those that were not H7 specific (14.16 ± 0.99; Fig. S4 e). Overall, we found that compared with the FRWs, the CDRs possessed a higher frequency of R mutations and a lower frequency of S mutations, suggestive of antigen-driven selection (Fig. S4 f). Interestingly, when we examined the cross-reactivity of H7-positive antibodies against the previous panel of influenza HA subtypes, we found that 12 of the H7-positive antibodies bound to at least one other influenza subtype and that three were panreactive with all subtypes (Fig. 5 f). In conclusion, by stimulating memory B cells in vitro with particulate H5- or H7-CPG, we were able to generate a high frequency of cross-reactive or even panreactive antibodies.

**Figure 6.**
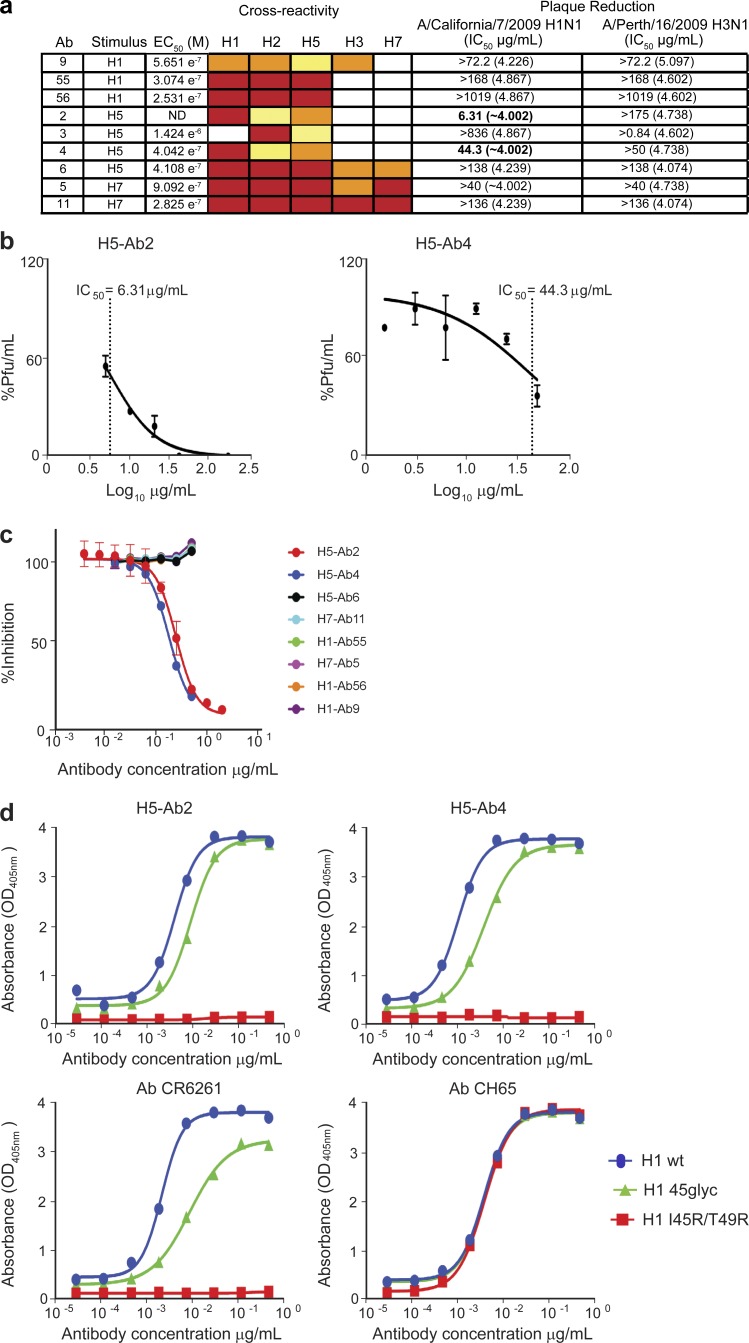
**Functional characterization of selected cross-reactive antibodies.** (a) Table showing the cross-reactivity, EC_50_ values, and plaque reduction assay IC_50_ values of selected recombinant antibodies. IC_50_ values are reported as micrograms per milliliter, with the respective positive control titer (in reciprocal log_10_ dilutions) in parentheses. Cross-reactivity was measured by ELISA; strong binding is coded in red (>2.0 OD_405_), intermediate in orange (1.0–2.0 OD_405_), and weak in yellow (0.3–1.0 OD_405_). (b) Plaque reduction assay showing the neutralization activity of IgGs H5-Ab2 and H5-Ab4, obtained from in vitro stimulation with particulate H5-CpG, on the A/California/7/2009 strain (H1 subtype). IC_50_ and logIC_50_ values were obtained by nonlinear regression fitting to a log (inhibitor) versus normalized response–variable slope model using Prism 6. Two replicate plates were used for each virus/test article experiment. Error bars represent SEM. (c) Competition ELISA showing the binding inhibition of the tested antibodies by the mouse mAb C179 to the HA stem. Dots represent the mean ± SD of two replicates. (d) ELISA reactivity of the indicated antibodies with wild-type HA from A/New Caledonia/20/1999 (H1 wt), H1 containing Arg substitutions that blocks access to the stem epitope, H1 delta stem (I45R/T49R), and H1 with an N-glycan insertion that also prevents access to the stem epitope, H1 delta stem (45glyc). The reference antibodies used are CR6261 IgG, a human stem-directed bnAb, and CH65 IgG, a human receptor-binding site (RBS)-directed bnAb. Dots represent the mean ± SD of two replicates.

Given our observation that these antibodies are highly cross-reactive, we wanted to determine whether any of the antibodies generated against the different HA proteins have the capacity to neutralize influenza virus. To this end, we chose nine mAbs (EC_50_ values in the micromolar range) based on their cross-reactivity for different influenza strains and tested their ability to inhibit either A/California/7/2009 (H1N1) or A/Perth/16/2009 (H3N2) by plaque reduction assay (Fig. 6 a). This assay revealed that two of these antibodies showed different degrees of inhibition (Fig. 6, a and b). The IC_50_ of the antibodies H5-Ab2 and H5-Ab4 (obtained after H5 stimulation) against H1N1 was 6.31 µg/ml (95% confidence interval, 5.391–7.395) and 44.43 µg/ml (95% confidence interval, 21.77–90.67), respectively, whereas none of the other seven antibodies showed any significant neutralization capacity (Fig. 6 b). These results suggest not only that our stimulation strategy leads to antibodies that can be highly cross-reactive, but more importantly, that some of them also have the capacity to neutralize virus.

To further characterize antibodies H5-Ab2 and H5-Ab4, we investigated their site of binding on HA. To this end, we performed a competitive ELISA with C179, a cross-neutralizing anti-HA stem antibody (Okuno et al., 1993). As shown in Fig. 6 c, the neutralizers H5-Ab2 and H5-Ab4 efficiently compete with C179 for binding to the A/California/7/2009 (H1N1) HA, indicating that these antibodies are binding to the HA stem. To further substantiate these findings, we evaluated the binding of these antibodies to different HA mutants by ELISA. We took advantage of wild-type HA from A/New Caledonia/20/1999 (H1 wt); a mutant HA containing arginine substitutions that block access to the stem epitope (H1 delta stem I45R/T49R), and a mutant with an N-glycan insertion that also prevents access to the stem epitope (H1 delta stem 45glyc). In this assay, the HA stem-binding antibody CR6261 and the HA head-binding antibody CH65 were used as controls. Although both antibodies bound to the positive control H1 wt, the binding of CR6261 to the I45R/T49R or 45glyc mutant proteins was compromised. On the other hand, the binding of Ab CH65 to both mutant variants was unaffected (Fig. 6 d). Both H5-Ab2 and H5-Ab4 lost the capacity to bind the I45R/T49R mutant (Fig. 6 d), confirming that these antibodies bind to the stem epitope. Moreover, these antibodies also showed reduced binding to the 45glyc mutant, further supporting the binding of H2-Ab2 and H5-Ab4 to the HA stem region (Fig. 6 d).

### Generation of HIV anti-gp120 antibodies by plasma cells derived from antigen-naive individuals

**Figure 7.**
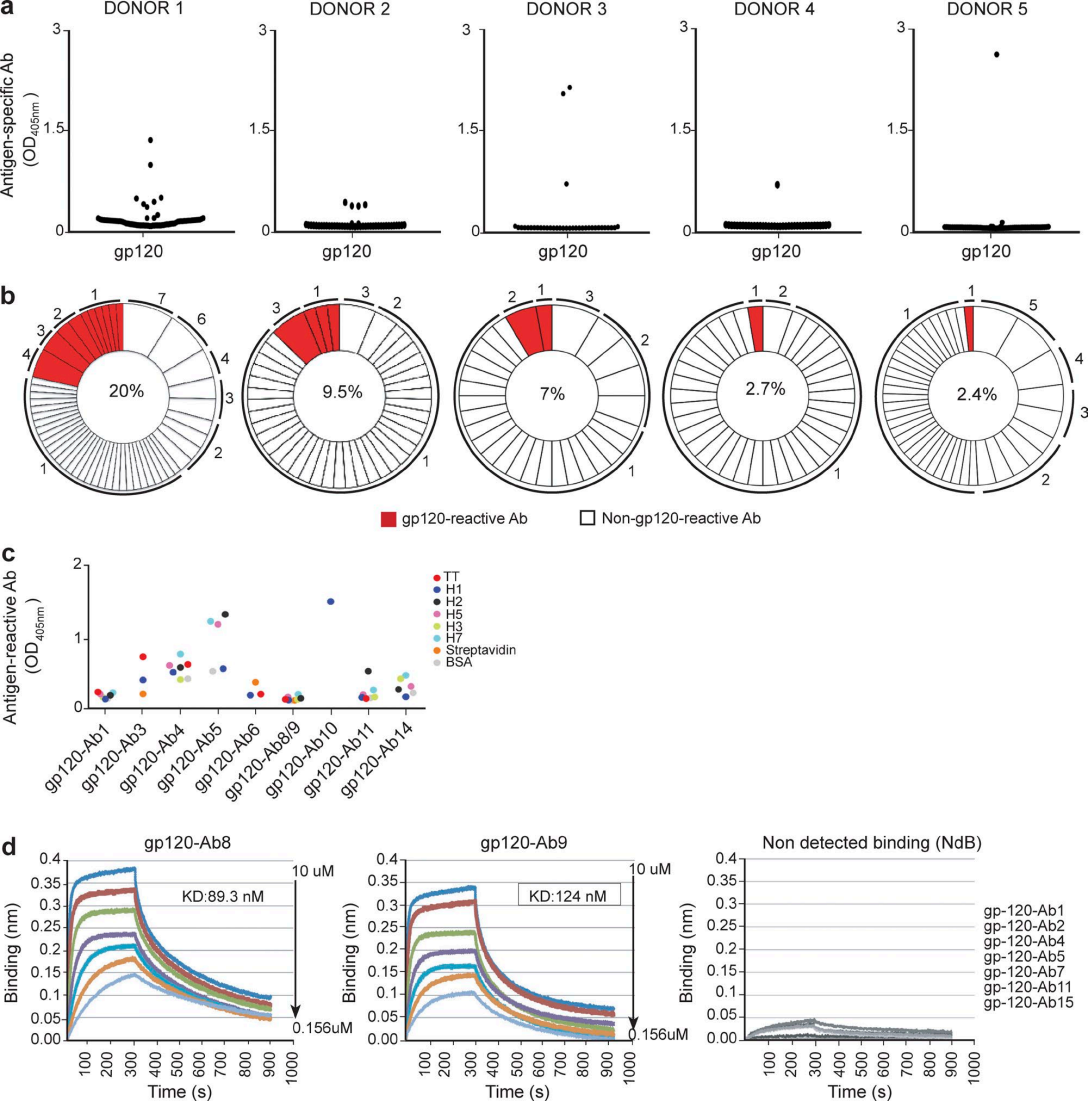
**Generation of gp120-specific antibodies from single sorted plasma cells.** (a) The specificity of the gp120 recombinant antibodies generated after stimulating memory B cells from five HIV-uninfected individuals was analyzed by ELISA. Cognate pairs of IgH and IgL genes were introduced into HEK293T cells by transient cotransfection, and culture supernatants were tested for the presence of gp120-specific IgGs. The mean IgG antibody concentration was 1 µg/ml. Cutoff value for a positive signal is taken as more than twofold above background. (b) Pie charts outlining the frequency of the gp120-specific clones. The number in the center of the pie chart indicates the percentage of the gp120-positive antibodies. Numbers outside the pie chart indicate the number of antibody gene sequences with identical IgH and IgL chain rearrangements. (c) Reactivity of the gp120-positive antibodies against a panel of foreign antigens was tested by ELISA. The cutoff value for positivity has been taken as more than twofold background. (d) Octet measurements of the kinetics of selected gp120-reactive antibodies to the recombinant wt 92BR gp-120 are shown. Arrows represent the serial dilution of the antibodies; K_d_ values are highlighted inside the charts.

So far, we have been able to use this in vitro vaccination booster to stimulate B cells that recognize TT, H1, H5, and H7. Although H5 and H7 are unlikely to have been experienced before by a healthy donor, these rare B cells might have originated as a result of previous H1 vaccination or infection. Therefore it was important to test the suitability of our approach using an antigen for which we are not relying on the preexistence of memory B cells from previous related-virus exposure. To this end, we stimulated memory B cells from five HIV-uninfected individuals with particulate gp120-CpG. We single-cell sorted, sequenced VH and VL, and expressed 257 antibodies. Eighteen of these antibodies were shown to be positive at recognizing recombinant gp120, as tested by ELISA and by flow cytometry (Figs. 7 a and S5 a). VH gene sequence alignment revealed antibodies with identical sequences in three of the five donors, suggesting in vitro B cell clonal expansion (Fig. 7 b). Analysis of somatic hypermutations in the rearranged VH gene segments showed 13 mutated VH sequences, with five to 34 mutations (16.54 ± 2.58; Table S7). This indicates that these B cells might have arisen from B cells that have previously been triggered by an unknown antigen. Additionally, we also identified five antibodies that contain from zero to three mutations from the closest germline (Table S8), which may reflect that the B cells that gave rise to these antibodies have not been affinity matured against gp120.

It was important to establish whether these antibodies exhibit a certain degree of specificity for gp120 or whether they were simply polyreactive. To this end, we tested nine representative gp120-reactive antibodies against a panel of different proteins using ELISA and flow cytometry (Figs. 7 c and S5 b). Although seven of the antibodies were able to bind to one or more proteins of the panel, antibodies gp120-Ab8 and gp120-Ab9 proved to be negative in this assay (Fig. 7 c). Moreover, biolayer interferometry (BLI) showed that gp120-Ab8 and gp120-Ab9 exhibited a measureable affinity of 89.3 and 124 nM to gp120, respectively, suggesting that they were specific to gp120 and were unlikely to be simply polyreactive (Fig. 7 d). These results show that our booster vaccination does not rely exclusively on the activation of memory B cells that originated from a related viral infection. Furthermore, because our methodology is independent of T cell help, it may be able to overcome tolerance mechanisms that would normally prevent these polyreactive specificities from responding to vaccination. Overall, our findings suggest that this method has the potential to be exploited as an important tool to study antigen immunogenicity in vitro.

## Discussion

Here, we introduce an in vitro booster vaccine strategy that allows antigen-specific human B cell activation and differentiation. This method exploits the exquisite capacity of the BCR to discriminate affinity. As a result, only B cells that are able to bind antigen with a certain avidity are able to internalize particulate-CpG. Synergistic BCR and TLR activation results in the induction of robust B proliferation and differentiation to plasma cells, together with antibody secretion in just a few days of in vitro culture. By using this approach, we were able to induce specific in vitro B cell responses for a variety of antigens such as TT, influenza, and gp120, mimicking an in vivo vaccine boost. This allowed the production of a large panel of human monoclonal antibodies within a few days. This in vitro vaccine boost strategy can be used not only as a way of studying antigen-specific antibody repertoires, but also to generate human mAbs.

Unlike that of T cells, efficient antigen-specific B cell activation in vitro has been difficult to achieve, because BCR engagement alone is insufficient to induce robust B cell activation. Indeed, B cells require second signals such as CD40 or TLR, and the presence of different interleukin cocktails, including IL-21, IL-2, IL-6, and IL-15 (Arpin et al., 1995; Bernasconi et al., 2002; Ettinger et al., 2005; Huggins et al., 2007). This type of stimulation has been used extensively to effectively activate polyclonal B cells (Corti et al., 2010; Smith et al., 2014). In contrast to CD40L stimulation, soluble CpG stimulation requires a high concentration, which forces entry through nonspecific uptake by the cells (Häcker et al., 1998), triggering a strong activation of B cells irrespective of BCR specificity. In contrast, an advantage of our method is that the binding of CpG to a nanosphere prevents nonspecific entry into B cells, so particulate-CpG can gain access to the TLR9 compartments only via antigen-specific BCR internalization. As a result, only those B cells that bind antigen receive the correct signals to proliferate and differentiate. Importantly, particulate anti-BCR stimulation alone is a poor inducer of proliferation and differentiation. Similarly, soluble CpG alone is sufficient to drive proliferation but not plasma cell differentiation. Stimulation of both BCR and TLR, however, results in robust proliferation, plasma cell differentiation, and antibody secretion. In the future, it will be important to define the precise signals involved in the induction of plasma cell differentiation.

TLR9 is expressed at high constitutive levels in memory B cells and can be directly triggered by CpG to induce B cells to proliferate and differentiate into antibody-secreting cells (Bernasconi et al., 2003). CpG stimulation has been suggested as one of the natural mechanisms to maintain serological memory (Bernasconi et al., 2002) and has been used as a polyclonal activator to expand EBV-transformed antigen-specific B cells (Traggiai et al., 2004). Earlier studies from our laboratory provided evidence for a platform with selective synergism between BCR and TLRs in stimulating B cells using particulates in mice, and the cross-linking of the two receptors leads to specific B cell proliferation and differentiation (Eckl-Dorna and Batista, 2009). Furthermore, synergisms of BCR and TLR9 have also been reported in the development of human autoimmune diseases (Leadbetter et al., 2002; Viglianti et al., 2003).

To optimize the conditions for in vitro stimulation, we first validated our system using the κ light chain as a model target antigen. We showed that only the integrated delivery of two signals, via both the BCR and the TLR, is able to drive the proliferation and differentiation of memory B cells into plasma cells, leading to an increased production of κ-bearing antibodies. In addition to the κ/λ proof of principle, we have successfully applied this approach to different antigens, demonstrating its robustness and reproducibility. We have shown that in vitro stimulation of memory B cells with particulate antigen-CpG (TT, influenza HA, gp120) selectively enriched the frequency of CD27^hi^/CD38^hi^ antigen-specific plasma cells despite the very low number of precursors circulating in peripheral blood and regardless of the nature of the pathogen-derived antigen chosen to coat the nanoparticles. These plasma cells were also able to secrete antibodies that were specific to the stimulating immunogen.

Analysis of the variable region sequences of the plasma cells recovered showed not only polyclonal responses, but also groups of identical variable regions. The repeated isolation of identical V(D)J sequences represents strong evidence of proliferation and differentiation of individual B cells in vitro; alternatively, it could reflect in vivo clonal size. We also found VDJ sequences with point mutations, which most likely represent sister cells that underwent in vivo somatic mutation and were individually stimulated in our in vitro system. Given the absence of T cell help and the short time in culture, it is unlikely that our system induces any somatic hypermutation. Future modifications to our antigen-specific in vitro B cell stimulation toward the induction of somatic mutation and affinity maturation represents a key next step in replicating a full immune response in culture.

In agreement with previously published data (Corti et al., 2011; Buricchi et al., 2013; Goff et al., 2013), we observed a degree of cross-reactivity between the antibodies retrieved from stimulation with H1N1, H5N1, H7N9, and other influenza HA subtypes, which led us to speculate that it may be possible to exploit this methodology to obtain antibodies against antigens that had not previously been encountered in vivo. This was further suggested by functional assays in which, of the nine antibodies tested, two demonstrated neutralizing activity against H1N1 HA. Indeed, we demonstrated that the two neutralizing antibodies identified in this study are specific to the stalk of HA. It has been shown that antibodies against the stem domain of HA are able to neutralize a wide spectrum of influenza virus strains and subtypes, because this domain is comparatively conserved (Ekiert et al., 2009; Corti et al., 2011; Dreyfus et al., 2012). Furthermore, three of the antibodies that were isolated from H1 stimulations, HA-Ab1, HA-Ab2, and HA-Ab3, are clonally related to two antibodies that have been recently identified as predominantly neutralizing antibodies developed upon H5 immunization in humans (Joyce et al., 2016). In the future, the combination of this methodology with high-throughput sequencing may prove to be an invaluable tool to study antigen immunogenicity in vitro.

So far, several powerful approaches have been used to isolate human monoclonal antibodies such as phage display, EBV immortalization, yeast display, and humanized animal models (McCafferty et al., 1990; Clackson et al., 1991; Arpin et al., 1995; Tiller et al., 2008; Boder et al., 2012; Shultz et al., 2012). However, all these methodologies rely on large-scale screening to identify only a few truly antigen-specific antibodies. Although more recently, antigen-specific B cell sorting and single-cell cloning have circumvented some of these issues, these approaches are still restricted to high-affinity antibody–antigen interactions (Franz et al., 2011). In contrast, the method reported here can overcome this barrier, as it does not rely on single BCR–antigen interactions but instead on the avidity of the BCR for the antigen arranged on the particle. As such, our approach allows antigen-specific isolation of Abs with K_d_ values ranging from 10^−9^ to 10^−12^ M for TT and influenza HA, and 10^−7^ to 10^−8^ M in the case of HIV gp120. Furthermore, our method induces the differentiation into antigen-specific plasma cells with the concomitant up-regulation of immunoglobulin genes compared with memory cells, thereby facilitating single-cell antibody cloning. Thus, our approach could be applied in future for the fast generation of human monoclonal antibodies, and it offers the possibility to interrogate antigen immunogenicity in vitro. This can be achieved in a matter of days, without the need of expensive GMP protein production or the involvement of patients in time-consuming clinical trials (Joyce et al., 2016). As such, our approach not only complements existing methodologies but also offers an alternative for an in vitro phase 0 trial. This will help in future vaccine development by accelerating the iterative immunogen design before entering into phase I clinical trial.

In summary, our study provides a framework for a novel and efficient in vitro platform for the selective stimulation of memory B cells from healthy donors, leading to proliferation and differentiation into plasma cells that produce antigen-specific antibodies, even in antigen-naive donors. Our methodology could have important applications in the production of therapeutic antibodies; specifically, it should allow their production within a shorter time frame in vitro and without the need for vaccination or blood/serum donation from recently infected or vaccinated individuals. In addition, our method offers the potential to evaluate and facilitate new vaccine development by allowing the efficient evaluation of any candidate target antigen.

## Materials and methods

### Reagents and antibodies

BSA and streptavidin were from Sigma-Aldrich. PBS was from Gibco. TT was supplied by the Statens Serum Institut. All recombinant influenza HA subtypes were from Sino Biological: H1N1 HA (A/California/04/2009; H1), H2N2 HA (A/Japan/305/1957; H2), H5N1 HA (A/Vietnam/1194/2004; H5), H3N2 HA (A/Perth/16/2009; H3) and H7N9 HA (A/Shanghai/1/2013; H7). H1N1 HA (A/New Caledonia/20/1999; H1 wt), the HA delta stem mutants H1 I45R/T49R and H1 45glyc, and the human monoclonal antibodies CR6261 and CH65 were provided by D. Lingwood. HIV-1 92BR-gp120 was provided by D.R. Burton (Scripps Institute, San Diego, CA). Human CpG ODN 2006 was from InvivoGen. IL-15 and IL-6 were from PeproTech. Antibodies used were as follows: goat anti–human IgG (FcΥ specific; Jackson ImmunoResearch Laboratories, Inc.); anti–human CD27-APC, anti–human CD38-FITC, CD138-PE, CD19-Percp Cy5.5, CD20-APC, CD80-PE-Cy5, HLA-DR-PE-Cy5, and anti–human κ-Ig biotin (eBioscience); anti-human CD86-Bv510, CCR10-PE, CD62L, CXCR4-PE-Cy5, CXCR5-APC, and anti–human λ-Ig biotin (BioLegend); and anti–human CD43-PE and anti–human IgD (Miltenyi Biotec).

### Nanoparticle coating

Streptavidin-coated 0.11-µm nanoparticles (Bangs Laboratories) were incubated with biotinylated CpG or biotinylated protein (anti-κ, anti-λ, BSA, TT, H1, H5, H7, gp120) overnight at 4°C. Coated nanoparticles were blocked with 3% PBS, IgG free, for 2 h at RT, washed twice with PBS + 0.5% BSA, IgG free, and resuspended in culture medium before sonication (117 VAC, 60 Hz, 0.5 A; Cole Parmer). Optimal stimulatory conditions were determined by increasing the amounts of CpG while keeping the amounts of anti-BCR/antigen constant. Coating efficiency was measured by flow cytometry.

### Human B cell activation

Leukocyte cones were obtained from the South East Regional Blood Transfusion Centre, UK. The health status of the donors for the lack of prior infection is based on mandatory screening for blood donations as described in the information contained within the issuers’ guidelines: http://www.transfusionguidelines.org/red-book/chapter-9-microbiology-tests-for-donors-and-donations-general-specifications-for-laboratory-test-procedures/9-1-general-requirements and http://www.transfusionguidelines.org/dsg/wb.

PBMCs were flushed from the leukocyte cones using a syringe and isolated using density gradient centrifugation (Lymphoprep; Nycomed). A mean of 15 × 10^8^ PBMCs were recovered per cone. Human CD27^+^ B cells were purified using Memory B Cell Isolation kit (Miltenyi Biotec) and stained with 10 nM CellTrace Violet (Thermo Fisher Scientific) according to the manufacturer’s protocol. After staining, purified human CD27^+^ B cells and particulates coated with biotinylated CpG and biotinylated anti-BCR antibody or antigen (in a ratio of 5,000 nanoparticles/cell) were cultured in a 96-well plate (200,000 purified memory B cells/well) at 37°C in 5% CO_2_ in complete medium: RPMI 1640 (Gibco) supplemented with 1 M MEM nonessential amino acids (Sigma-Aldrich), 1 M Hepes, 1 M GlutaMAX, 10 U/ml penicillin-streptomycin, 50 µM β_2_-mercaptoethanol, 10% heat-inactivated FBS (Thermo Fisher Scientific), and 10 ng/ml IL-15 in a 96-well plate. After 3 d, 10 ng/ml IL-6 was added, and cells were cultured for another 3 d. After a total of 6 d of stimulation, B cells were harvested to assess proliferation and differentiation by flow cytometry. Supernatant was collected at day 6 from cultures of anti-BCR stimulated cells for subsequent ELISA.

### Flow cytometry and single-cell sorting

Cells were harvested after 6 d of stimulation and blocked with an FcR blocking reagent (Miltenyi Biotec). After washing with FACS buffer (PBS, 1% FBS, 1% BSA), cells were stained with anti–human CD27-APC and anti–human CD38-FITC and washed again before being analyzed on a BD FACSAria III. Flow cytometry data were analyzed using FlowJo version 10.0.7 (Tree Star). Alternatively, CD27^+^/CD38^+^ plasma cells were sorted by FACSAria III into individual wells of 96-well PCR plates. Plates were sealed with foil film (Bio-Rad) and immediately frozen on dry ice before storage at −80°C.

### ELISA

96-well plates (Corning) were coated with 1 µg/ml anti–human IgG or the following proteins: TT, BSA (referred as protein control), streptavidin, H1, H2, H3, H5, H7, H1 wt, H1 I45R/T49R, H1 45glyc, or gp120 and incubated overnight at 4°C. Plates were washed with PBS with 0.01% Tween (PBS-T; Sigma-Aldrich) and blocked with a protein-free buffer (Thermo Fisher Scientific) for 1 h at RT. After blocking the plates, samples were added and incubated at RT for 2 h. After rinsing with PBS-T, plates were incubated with anti–human IgG-biotin or anti–human κ-biotin or λ-biotin at a concentration of 1 µg/ml at for 1 h followed by ExtrAvidin alkaline phosphatase (Sigma-Aldrich) at RT for 1 h. Plates were developed by adding phosphatase substrate (SigmaFas *p*-nitrophenyl phosphate substrate; Sigma-Aldrich). OD was determined at 405 nm with a Spectra-max 190 microplate reader (Molecular Devices). For the competitive ELISA, a twofold serial dilution of the test antibodies was premixed with 0.5 µg/ml of the reference mouse anti–human H1 stem antibody (C179; Takara Bio, Inc.) on a noncoated U-bottom 96-well plate (Costar). The premixed antibodies were then transferred to a H1 (A/California/04/2009)-coated plate and incubated for 1 h at RT. After washing and adding 0.5 µg/ml anti-mouse secondary antibody coupled to biotin (goat anti–mouse IgG biotin, human adsorbed; Southern Biotech), the ELISA was continued as previously described.

### Transmission electron microscopy

B cell populations were sorted into 4% (vol/vol) formaldehyde in 0.1 M phosphate buffer (final concentration 2%), centrifuged gently (650 *g*) for 5 min, and resuspended in 2.5% (vol/vol) glutaraldehyde/4% (vol/vol) formaldehyde in 0.1 M phosphate buffer for 30 min at RT. Cells were postfixed with 2% osmium tetroxide/1.5% potassium ferricyanide for 1 h at 4°C and stained with 1% thiocarbohydrazide for 20 min, followed by 2% aqueous osmium for 30 min and 1% aqueous uranyl acetate overnight at 4°C. The pellets were then stained with Walton’s lead aspartate for 30 min before being dehydrated stepwise through an ethanol series (10 min each at 30%, 50%, 70% 90%, 2× 100% on ice, and 10 min 100% at RT) and embedded in Durcupan ACM (Sigma-Aldrich). Blocks were sectioned using a UC6 ultramicrotome (Leica Microsystems) and picked up on 150-mesh hexagonal copper grids (Gilder Grids). Sections were viewed using a 120-kV Tecnai G2 Spirit transmission electron microscope (FEI Company), and images were captured using an Orius CCD (Gatan). All cells within several randomly selected grid areas were imaged (60–100 cells per condition using four donors) and quantified based on the structure of their ER (expanded or nonexpanded).

### ELISPOT

ELISPOT plates (EMD Millipore) were coated with 5 µg/ml TT or H1. After overnight incubation at 4°C, plates were washed three times with sterile PBS and blocked with RPMI and 10% FBS at RT. Plates were washed again, and stimulated B cells were added to the plates in complete medium. After incubation at 37°C 5% CO_2_ for at least 16 h, plates were washed with PBS-T (Sigma-Aldrich), and 100 µl of 1 µg/ml goat anti–human IgG-biotin was added to each well. After 1 h, plates were washed with PBS-T, incubated with ExtrAvidin alkaline phosphatase (Sigma-Aldrich,) and developed by adding SigmaFast BCIP/NBT (Sigma-Aldrich) substrate. The reaction was stopped by washing with distilled water. Plates were air-dried, and the spots were imaged and counted with the ELISPOT reader (CTL-ImmunoSpot).

### Single-cell RT-PCR and antibody cloning

First-strand cDNA from single cells was synthesized in the original 96-well plate with SuperScript III reverse transcription (Invitrogen) using oligo-dT. Nested PCR reactions and expression vector cloning were performed as previously described (Tiller et al., 2008). In brief, cDNA was synthesized using 150 ng random hexamer primer (pd(N)6; GE Healthcare), 0.5 µl of 10 mM of each nucleotide dNTP-Mix (Invitrogen), 1 µl of 0.1 M dithiothreitol (Invitrogen), 0.5% vol/vol Igepal CA-630 (Sigma-Aldrich), 4 U RNAsin (Promega), 6 U Prime RNase Inhibitor (Eppendorf), and 50 U Superscript III reverse transcription (Invitrogen) at the following temperatures: 42°C for 10 min, 25°C for 10 min, 50°C for 60 min, and 94°C for 5 min. IgH, Igλ, and Igκ V gene transcripts were amplified independently by nested PCR starting from 3.5 µl cDNA as template. Each round of PCR was performed for 40 cycles at 94°C for 30 s, 58°C (IgH/Igκ) or 60°C (Igλ) for 30 s, 72°C for 55 s (first PCR) or 45 s (second PCR). IgH, Igλ, and Igκ PCR products were purified using Qia-Quick 96 PCR Purification kit (QIAGEN) and digested with the respective restriction enzymes AgeI, SalI, and XhoI (all from New England Biolabs, Inc.). Digested PCR products were purified and ligated into human Igγ1, Igκ, and Igλ expression vectors. Competent *Escherichia coli* DH10B bacteria (Clontech) were transformed at 42°C with 3 µl of the ligation product. Colonies were screened by PCR using 5′ Ab sense as forward primer and 3′ IgG internal, 3′ Cκ494, or 3′ Cλ as reverse primer. Plasmid DNA was isolated from 4-ml bacteria cultures grown for 16 h at 37°C in Terrific Broth (Difco Laboratories) containing 75 µg/ml ampicillin (Sigma-Aldrich) using QIAprep Spin columns (QIAGEN). All kits and reagents were used according to the manufacturers’ instructions.

### Antibody sequence analysis

DNA sequencing was performed on an Applied Biosystems 3730xl DNA analyzer. Sequences were compared with known germline genes and assigned IGHV and IGHJ germline sequences based on the highest percentage of sequence homology using IMGT (http://www.imgt.org) and IgBLAST (http://www.ncbi.nlm.nih.gov/igblast/) databases. The variable heavy and light chain antibody genes were analyzed for gene usage, mutations within FWR1-3 and CDR1/2, and length of CDR3. Replacement and silent mutations in FWR1-3 and CDR1/2 were determined and normalized to the respective length of each region as defined by IMGT and IgBLAST.

### Recombinant antibody production and purification

Heavy and light chain plasmids were cotransfected into exponentially growing HEK293T cells (CRL-11268; ATCC) by electroporation with an Amaxa 4D Nucleofector (Lonza) according to the manufacturer’s protocol. After 3 d, supernatants were harvested and analyzed by ELISA for recombinant antibody production. Antibody names are codified by the antigen stimulus followed by the antibody number (for example, H5-Ab2). Recombinant antibody concentrations were determined by ELISA as previously described (Tiller et al., 2008). For neutralization assays, recombinant antibodies were purified from supernatants of transfected HEK293T cells cultured for 3 d in serum-free Opti-MEM Medium (Thermo Fisher Scientific). Cells debris was removed by centrifugation at 1600 rpm for 10 min, and the human IgG was purified by AKTA Start, using a protein A column (HiTrap MabSelect SuRe; GE Healthcare) according to the manufacturer’s instructions.

### Analysis of antibody affinities

The kinetic interaction of mAb with recombinant HA protein was determined by surface plasmon resonance (SPR) using a Biacore 2000 system or by biolayer interferometry using Fortebio Octet. For Biacore, purified HA protein was diluted to 30 µg/ml in 10 mM sodium acetate, pH 4.5, and covalently immobilized at 5 µl/min by amine coupling to the dextran matrix of a CM5 sensor chip (Biacore), with a target density of 1,200 response units. Unreacted active ester groups were blocked with 1 M ethanolamine. All five purified influenza-specific mAbs, at concentrations ranging from 5 to 500 nM in HBS/Tween-20 buffer (Biacore), were injected over the immobilized HA protein or reference cell surface. Association rate (K_on_), dissociation rate (K_off_), and equilibrium dissociation constant (K_d_) were calculated by aligning the binding curves globally to fit a 2:1 Langmuir binding model using BIAevaluation v4.1. Octect was performed in black 96-well plates (Nunc F96 MicroWell Plates; Thermo Fisher Scientific) in a total working volume for samples or buffer of 200 µl per well. Before each assay, streptavidin biosensor tips (ForteBio Octect) were prewetted in 200 µl of 1× kinetic buffer for at least 10 min. Afterward, streptavidin biosensor tips were loaded with biotinylated antigen in 1× PBS (12.3 µM), followed by an additional equilibration step (120 s). Subsequently, association of biotinylated antigen with antibody over a concentration range of 60, 30, 15, 7.5, 3.75, 1.875, 0.094, and 0 nM in 1× kinetic buffer was conducted. Association at each concentration was performed for 300 s. Finally, the dissociation was monitored in 1× kinetic buffer for 600 s. Results were plotted showing dissociation from 300 to 600 s.

### Plaque reduction assay

The influenza plaque assay was performed using an adaptation of a previously described plaque assay method (Matrosovich et al., 2006). A series of six twofold dilutions of each antibody (and a polyclonal antiserum positive control specific for each virus) was prepared using DMEM containing 530.45 USP/NFP Trypsin *N*-tosyl-l-phenylalanyl chloromethyl ketone (TPCK). These dilutions were then incubated at a 1:1 (vol/vol) ratio with 250 µl of 100 PFU/ml of either A/Perth/16/2009 or A California/7/2009 at 37°C/5% CO_2_ for 1 h, with the samples regularly inverted to ensure homogeneity. Six-well plates of MDCK cells (seeded at 5 × 10^5^ cells/ml the day before the assay) were washed twice with PBS, and 400 µl of the virus/mAb mix was overlaid onto individual wells, one well per dilution. After a further 1-h incubation, cells were overlaid with 2.5 ml Avicel solution (2.4% Avicel in DMEM containing 530.45 USP/NFP Trypsin TPCK and 0.45% sodium bicarbonate) and incubated at 35°C 5% CO_2_ for 2 d (A/California/7/2009) or 3 d (A/Perth/16/2009). The plates were then washed twice using PBS and subsequently fixed and stained using formalin and crystal violet solution (7.14% formalin and crystal violet, 14.29% ethanol in H_2_O). Each mAb was tested in triplicate. A back titration plaque assay was also performed for each virus to confirm that the virus titers were within acceptable parameters. The titer determined from this back-titration was subsequently used to determine the IC_50_ calculations. Plaques were visually counted, and IC_50_ data were calculated using Prism v5 (GraphPad Software).

### Quantitative PCR

After 6 d of stimulation, CD27^hi^/CD38^int^ and CD27^hi^/CD38^Hi^ cells were sorted and lysed for RNA extraction using the Mag-max 96 total RNA isolation kit (Thermo Fisher Scientific) and reverse transcribed using random hexamers and the transcriptor first-strand cDNA kit from Roche. Quantitative real-time PCR was performed using the ViiA 7 system, and cDNA levels were detected using power SYBR green (Thermo Fisher Scientific) and normalized to GAPDH for each sample. All reported values were then further normalized to memory B cells. The oligonucleotides used for Q-PCR were as follows: BCL6 forward (5′-AAGGGTCTGGTTAGTCCACAG-3′) and reverse (5′-GGTCACACTTGTAGGGTTTGTC-3′); PRDM1 forward (5′-CAACAACTTTGGCCTCTTCC-3′); PRDM1 reverse (5′-GCATTCATGTGGCTTTTCTC-3′); PAX-5 forward (5′-GGAGGAGTGAATCAGCTTGG-3′); PAX-5 reverse (5′-GGCTTGATGCTTCCTGTCTC-3′); IRF-4 forward (5′-ACCGAAGCTGGAGGGACTAC-3′) and reverse (5′-GTGGGGCACAAGCATAAAAG-3′); and XBP-1 forward (5′-TCACCCCTCCAGAACATCTC-3′) and reverse (5′-AAAGGGAGGCTGGTAAGGAA-3′). Differences in gene expression between CD27^hi^/CD38^int^ and CD27^hi^/CD38^hi^ were calculated with a *t* test, and the statistical significance was determined using the Holm–Sidak method to correct for multiple comparisons, with α = 5.000% (P < 0.05).

### Cell stimulation and immunoblotting

Purified memory B cells were equilibrated in complete medium at 37°C for 10 min, and prewarmed particulates coated with biotinylated CpG and biotinylated anti BCR or biotinylated anti-BCR were added at a ratio of 5,000 particulates per B cell. Soluble CpG was added at a final concentration of 1 µg/ml for the indicated time. Cells were lysed in 1% NP-40 buffer and analyzed by SDS-PAGE followed by immunoblotting with indicated antibodies. Anti–phospho-Lyn (Y507), anti–phospho-ERK, anti–phospho-Akt (Ser473), anti–phospho-Syk, anti–phospho-PLCϒ2, anti–phospho-CD19, anti–phospho-SHP-1, and anti-ERK were all from Cell Signaling Technology. The density of the bands was quantified by densitometry, corrected for background, normalized to the density of the actin band in the same sample, and made relative to the unstimulated zero time point for each condition.

### Statistics

Differences in CDR3 length and V gene mutations were calculated by paired two-tailed Student’s *t* test. Differences were considered to be statistically significant at values of P ≤ 0.05. EC_50_ values were calculated using nonlinear regression (curve fit), parameter: log (agonist) vs. normalized response − variable slope. IC_50_ and logIC_50_ values were calculated using nonlinear regression (curve fit), parameter: log (inhibitor) vs. normalized response − variable slope. All statistical analyses were performed with Prism v6.0a and h.

### Online supplemental material

Fig. S1 provides a comparison of in vitro stimulation of memory B cells obtained from fresh versus frozen PBMCs. Fig. S2 describes the phenotype of the CD27^high^/CD38^int^ and CD27^high^/CD38^high^ cell populations and gene expression of transcription factors involved in plasma cell differentiation. Fig. S3 shows an analysis of the sequence of TT-specific antibodies. Fig. S4 shows a sequence analysis of HA-specific antibodies. Fig. S5 shows an analysis of the reactivity of the gp120 binding antibodies by flow cytometry. Table S1 lists unique antigen-specific antibody sequences isolated from single plasma cells obtained after stimulation of memory cells of healthy individuals with particulate TT-CpG. Table S2 shows biolayer interferometry to determine K_on_, K_off_, and K_d_ of selected TT-specific antibodies binding to immobilized antigen. Table S3 shows unique antigen-specific antibody sequences isolated from single plasma cells obtained after stimulation with particulate H1-CpG. Table S4 shows SPR measurements of K_on_, K_off_, and K_d_ of selected H1-specific antibodies binding to immobilized antigen. Table S5 shows characteristics of the unique antigen-specific antibody sequences isolated from single plasma cells obtained after stimulation with particulate H5-CpG. Table S6 shows characteristics of the unique antigen-specific antibody sequences isolated from single plasma cells obtained after stimulation with particulate H7-CpG. Table S7 shows characteristics of the unique antigen-specific antibody sequences isolated from single plasma cells obtained after stimulation with particulate gp120-CpG. Table S8, included as an Excel file, shows sequences of all antibodies isolated in this study.

## Supplementary Material

Supplemental Materials (PDF)

Table S8 (Excel file)
