# Supplementary material for: Novel in vitro booster vaccination to rapidly generate antigen-specific human monoclonal antibodies
Source: J Exp Med. 2017 Aug 7;214(8):2471–90. doi: 10.1084/jem.20170633 (PMC5551578; doi:10.1084/jem.20170633)
Supplement: Supplemental Materials (PDF) [file JEM_20170633_sm.pdf]

The Journal of Experimental Medicine

## SUPPLEMENTAL MATERIAL

Sanjuan Nandin et al., https://doi.org/10.1084/jem.20170633

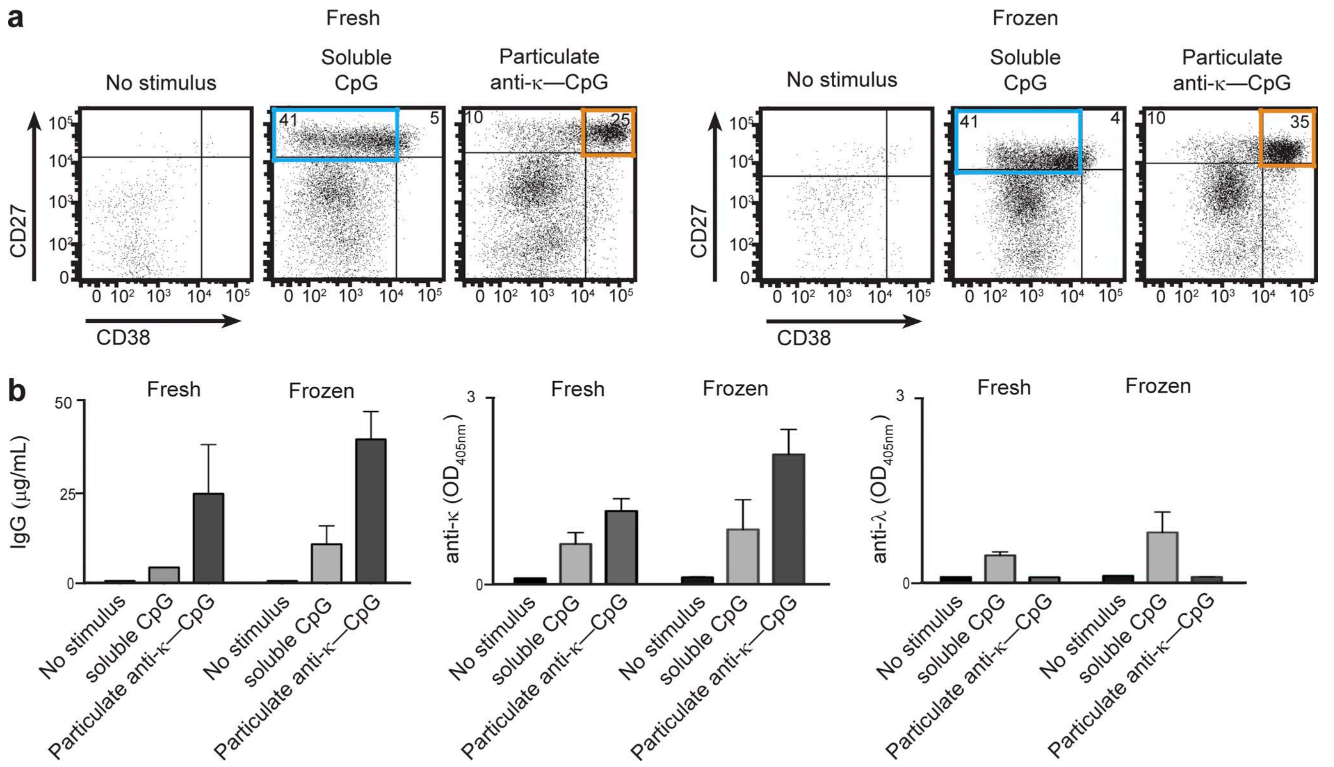


Figure S1. **Comparative in vitro stimulation of memory B cells isolated from fresh and cryopreserved PBMCs.** (a) CellTrace Violet–labeled memory B cells were cultured for 6 d with particulate anti–BCR-CpG, particulate anti-BCR, or soluble CpG. Numbers in the plots indicate the percentage of plasmablasts and plasma cells based on the expression of CD27/CD38 on proliferating cells. (b) Concentrations of IgG antibodies secreted in the culture supernatant and the presence of κ- or λ-bearing antibodies were determined by ELISA. Left, mean ± SD IgG concentrations. Middle and right, ELISA measurements of κ- and λ-chain Igs, respectively. Results represent mean $OD_{405}$ values ± SD of duplicate samples.

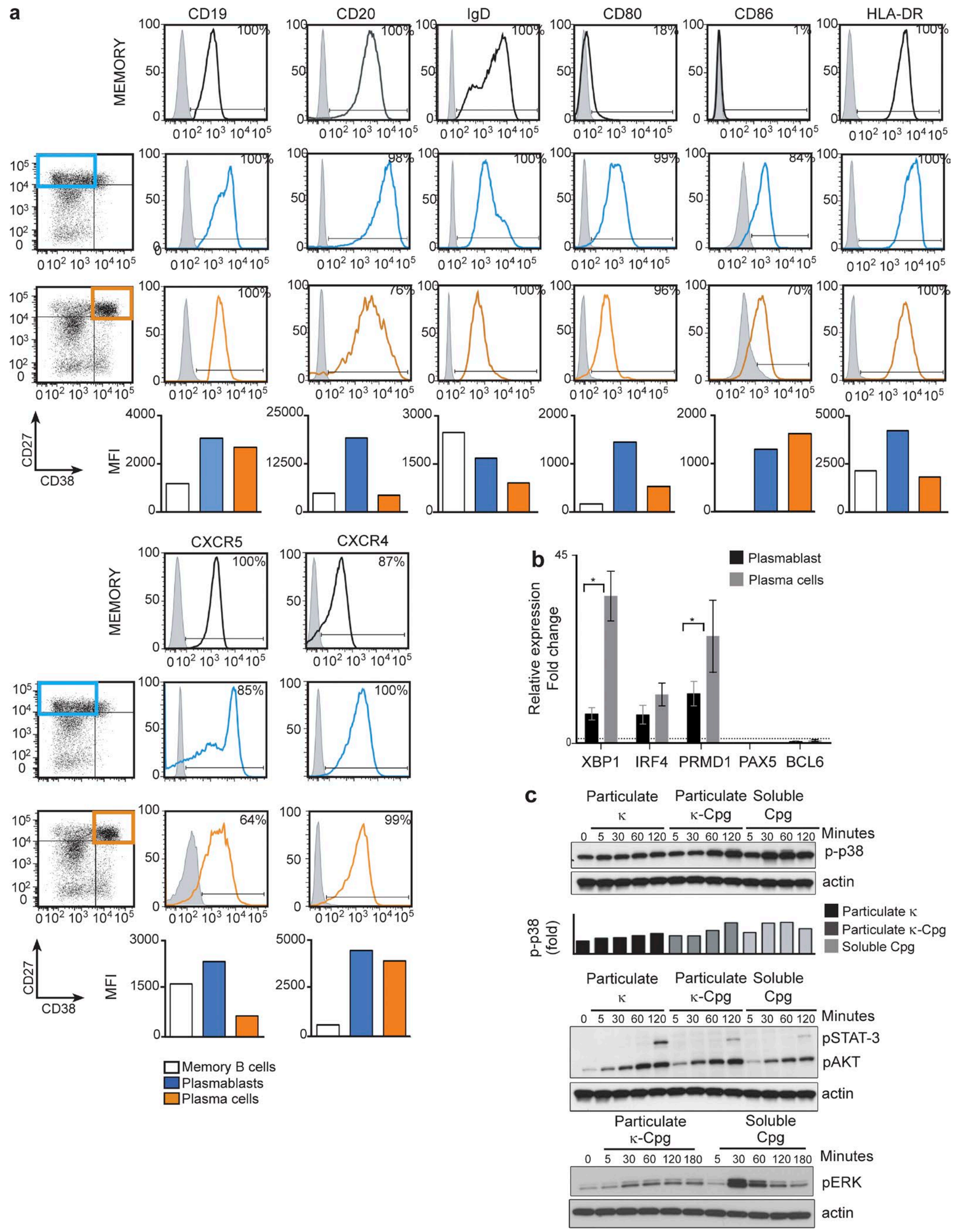


Figure S2. **Phenotype of the CD27$^{high}$/CD38$^{int}$ and CD27$^{high}$/CD38$^{high}$ cell populations and gene expression of transcription factors involved in plasma cell differentiation.** (a) The cell surface phenotype was analyzed by gating on plasmablasts (blue gate) generated after soluble CpG stimulation or plasma cells (orange gate) generated after particulate anti–κ-CpG stimulation. Phenotype of the memory B cells before stimulation is included. Data from one representative experiment of four are shown. Gray histograms represent the corresponding isotype control. Numbers in panels indicate the percentage of positive cells. The bar charts represent the mean fluorescence intensity (MFI) for the indicated surface markers. (b) Purified memory B cells and sorted CD27$^{hi}$/CD38$^{int}$ and CD27$^{hi}$/CD38$^{high}$ cells were evaluated for expression of XBP1, IRF4, PRMD1, PAX5, and BCL6 by real-time PCR. mRNA expression was normalized against the GAPDH gene and is presented as fold difference of the memory B cells (dashed line). Significant differences in gene expression between CD27$^{hi}$/CD38$^{int}$ and CD27$^{hi}$/CD38$^{high}$ cells are indicated (*t* test, statistical significance determined using the Holm–Sidak method to correct for multiple comparisons, with $\alpha$ = 5.000%; $P < 0.05$). Data are mean ± SD of gene expression determined in six experiments. (c) Purified B cells were stimulated with either particulate κ or particulate κ-CpG (5,000 nanoparticles/cell) of soluble CpG (1 µg/ml). Samples were harvested at the indicated times, and equal amounts of whole-cell lysates were subjected to SDS-PAGE and examined for phosphorylation of p38, STAT-3, AKT, and ERK. Chart shows the densitometry analysis of the bands relative to actin. Data from one representative experiment of six are shown.

**a**

```
                                <---------------------------------------------  FR1 - IMGT  -----------------------------
                                1               5                   10                  15                  20
                                 Q   V   Q   L   V   Q   S   G   A       E   V   K   K   P   G   S   S   V   K   V   S   C
L22583 Homsap IGHV1-69*06 F     cag gtg cag ctg gtg cag tct ggg gct ... gag gtg aag aag cct ggg tcc tcg gtg aag gtc tcc tg

TT-Ab3                          --- --- --- --- --- --- --- --- --- ... --- --- --- --- --- --- --- --- --- --- --- --- --
                                                     E                       M
TT-Ab4                          --- --- --- --- --- g-- --- --- --- ... --- a-- --- --- --- --- --- --- --- --- --- --- --

                                ------------>  _________________  CDR1 - IMGT  ______________  <-------------------------
                                      25                  30                  35                  40                  45
                                   K   A   S   G   G   T   F                   S   S   Y   A   I   S   W   V   R   Q   A
L22583 Homsap IGHV1-69*06 F     c aag gct tct gga ggc acc ttc ... ... ... ... agc agc tat gct atc agc tgg gtg cga cag gcc
                                       V               A                       N
TT-Ab3                          - --- -t- --- --- --- g-- --- ... ... ... ... -a- --- --- --- --- --- --- --- --- --- ---
                                   R   M               K                       N   N           M
TT-Ab4                          - -g- atg --- --- --- -ag --- ... ... ... ... -a- -a- --- --c --g --t --- --- --- --- ---

                                 FR2 - IMGT  -------------------------> ______________  CDR2 - IMGT  __________  <---------
                                                50                  55                  60                  65
                                 P   G   Q   G   L   E   W   M   G   G   I   I   P   I           F   G   T   A   N   Y   A
L22583 Homsap IGHV1-69*06 F     cct gga caa ggg ctt gag tgg atg gga ggg atc atc cct atc ... ... ttt ggt aca gca aac tac gc
                                                                                                 L   R                   T
TT-Ab3                          --- --- --- --- --- --- --- --- --c --- --- --- --- --- ... ... --g c-- --- --- --- --- a-
                                                             V                       V           L
TT-Ab4                          --- --- --- --a --- --a --- g-- --- --- --- --- --- g-a ... ... c-- --g --- --- --- --- --

                                ------------------------------------------------------------  FR3 - IMGT  ---------------
                                      70                  75                  80                  85                  90
                                   Q   K   F   Q       G   R   V   T   I   T   A   D   K   S   T   S   T   A   Y   M   E
L22583 Homsap IGHV1-69*06 F     a cag aag ttc cag ... ggc aga gtc acg att acc gcg gac aaa tcc acg agc aca gcc tac atg gag
                                   E                                       N                       N
TT-Ab3                          - g-- --- --- --- ... --- --- --- --- --- -a- --- --- --- --- --- -a- --- --- --- --- --a
                                                       D           A                               N
TT-Ab4                          - --- --- --- --- ... -a- --- --- g-- --a --- --- --- --- --- --- -a- --- --- --- --- ---

                                ------------------------------------------------------>  CDR3 -  IMGT
                                                95                  100             104
                                 L   S   S   L   R   S   E   D   T   A   V   Y   Y   C   A   R
L22583 Homsap IGHV1-69*06 F     ctg agc agc ctg aga tct gag gac acg gcc gtg tat tac tgt gcg aga ga
                                         K                                                       V   W   G   T   T   V   T
TT-Ab3                          --- --- -aa --- --- --- --- --- --- --- --- --- --- --- --- --- -ta tgg ggg act acg gtg ac
                                                         D                                       V   W   G   T   V   V   T
TT-Ab4                          t-- --- --- --- --- --- --c --- --- --- --- --- --- --- --- c-- -ta tgg ggg act gtc gtg ac
```

**b**

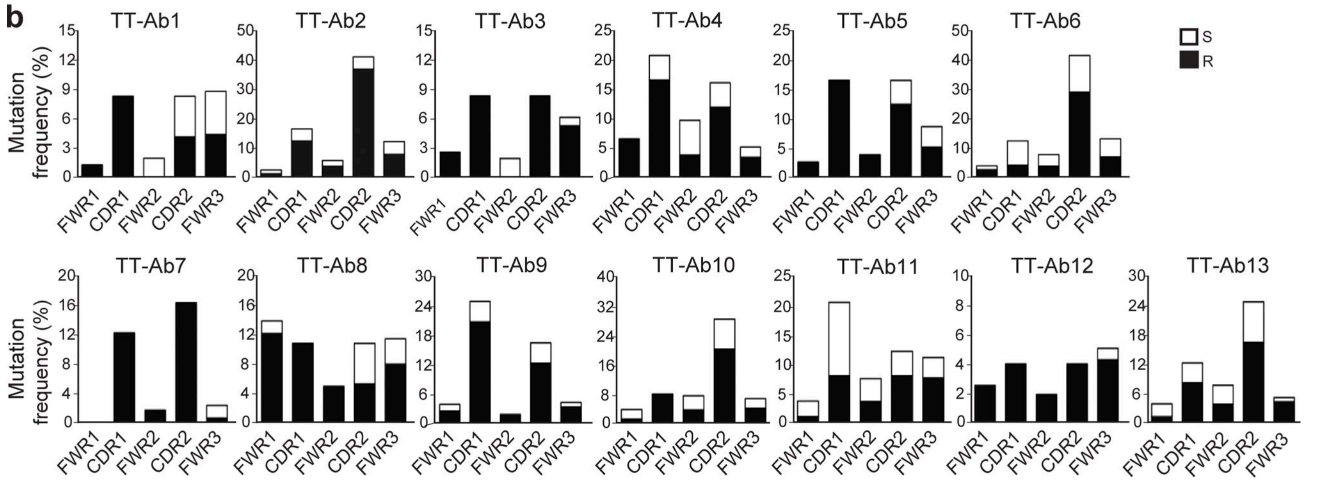


Figure S3. **Sequence analysis of TT-specific antibodies.** (a) Sequence alignment of clonally related TT-specific antibodies. VH sequences from two TT-specific antibodies with the same VH and VL gene usage were aligned against the germline gene counterpart (IGHV1-69*06). Dashes represent identity with the germline sequence. Replacement amino acid changes observed are highlighted in pink and blue. (b) Frequency of replacement (R, black) and silent (S, white) mutations in the CDRs and FWRs of the TT-specific antibody VH genes.

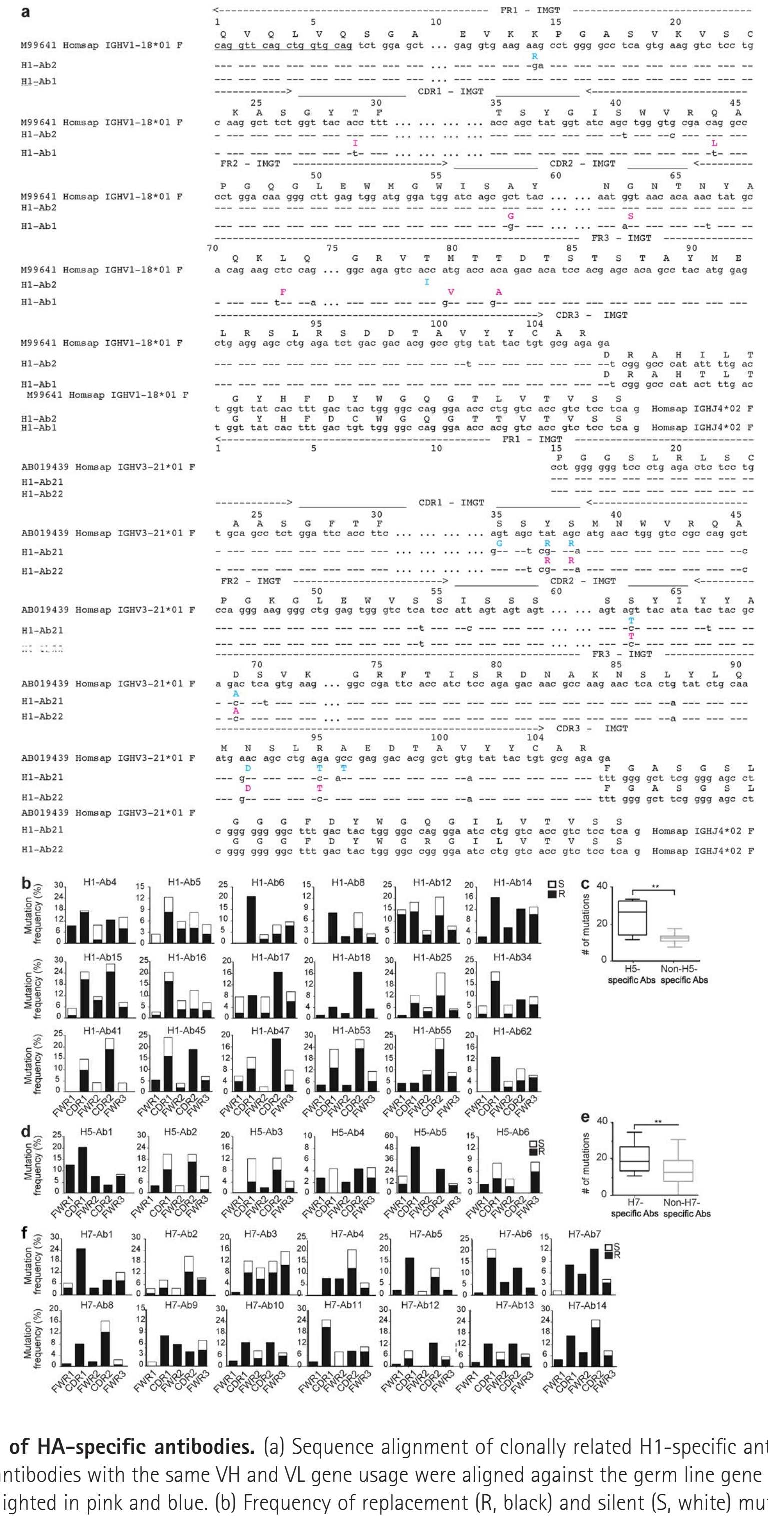

Figure S4. **Sequence analysis of HA-specific antibodies.** (a) Sequence alignment of clonally related H1-specific antibodies. VH sequences from two different clones of H1-specific antibodies with the same VH and VL gene usage were aligned against the germ line gene counterpart. Replacement amino acid changes observed are highlighted in pink and blue. (b) Frequency of replacement (R, black) and silent (S, white) mutations in the CDRs and FWRs of the VH genes. Representative examples of H1-specific antibodies are shown. (c) Comparison of the absolute numbers of somatic mutations in the VH genes encoding five H5-reactive and eight nonreacting antibodies. Boxes represent the percentile range (25–75%), the horizontal bar indicates the median, and whiskers extend to the highest and lowest data points. Two-tailed P values were calculated with an unpaired *t* test (**, P < 0.05). (d) Frequency of replacement (R, black) and silent (S, white) mutations in the CDRs and FWRs of VH genes of H5-reactive antibodies VH genes. (e) Comparison of the absolute numbers of somatic mutations in the VH genes encoding 14 H7-reactive and 57 nonreactive antibodies. Boxes represent the percentile range (25–75%), the horizontal bar indicates the median, and whiskers extend to the highest and lowest data points. Two-tailed P values were calculated with unpaired *t* test (**, P < 0.05). (f) Frequency of replacement (R, black) and silent (S, white) mutations in the CDRs and FWRs of the VH genes of the H7-reactive antibodies.

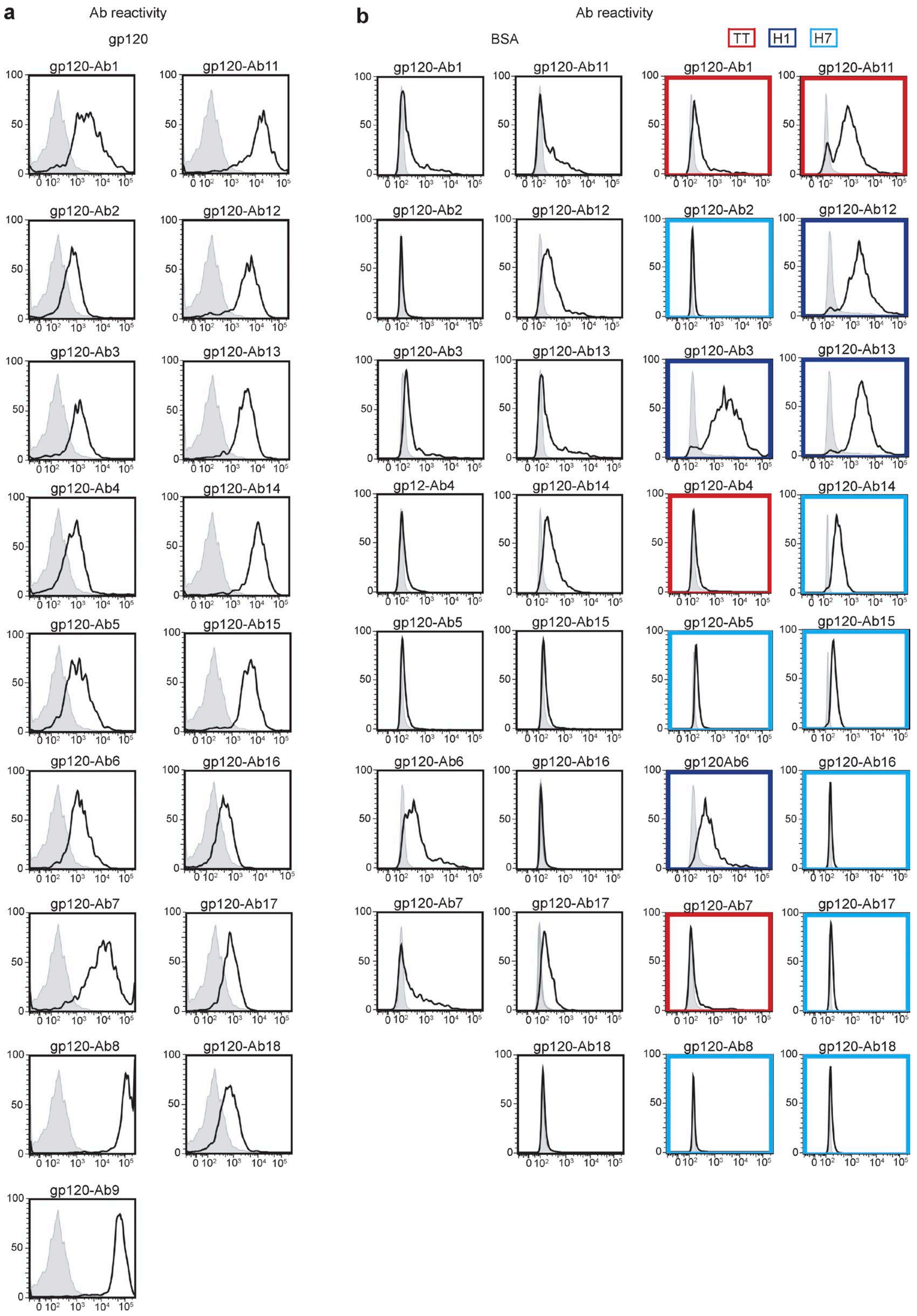


Figure S5. **Reactivity of the gp120-binding antibodies by flow cytometry.** (a) Confirmation of gp120 specificity of the recombinant antibodies screened by ELISA. Streptavidin nanoparticles coated with biotinylated gp120 were incubated with the generated antibodies, and reactivity was detected with an Fc-specific, PE-labeled anti–human IgG. Gray histograms, staining controls. (b) Reactivity of the gp-120-binding antibodies was tested against a panel of foreign antigens (BSA, TT, H1, H7) following the protocol described in the text.

Table S1. **Characteristics of the unique antigen-specific antibody sequences isolated from single plasma cells obtained after stimulation of memory cells of healthy individuals with particulate TT-CpG**

| Ab | VH name | JH name | DH name | CDR3 length | CDR3 sequence | VL name | JL name | CDR3 length | CDR3 sequence |
|---|---|---|---|---|---|---|---|---|---|
| | | | | *aa* | | | | *aa* | |
| TT-Ab1 | IGHV1-18*01 | IGHJ4*02 | IGHD3-22*01 | 15 | CARGLRHYHDGSPLEYW | IGKV3-20*01 | IGKJ5*01 | 9 | CQLYGNSLITF |
| TT-Ab2 | IGHV1-46*01 | IGHJ4*02 | IGHD5-24*01 | 11 | CAGGGQRFTFDYW | IGLV2-23*02 | IGLJ2*01 | 10 | CCSYAGNSNVIF |
| TT-Ab3 | IGHV1-69*06 | IGHJ6*02 | IGHD4-17*01 | 24 | CARVWGTTVTKGPSQIKYYYYGMDVW | IGKV1-39*01 | IGKJ4*01 | 9 | CQQSYSTPLTF |
| TT-Ab4 | IGHV1-69*06 | IGHJ6*01 | IGHD4-17*01 | 24 | CARVWGTVVTQGPSQVQYNHYMDVW | IGKV1-39*01 | IGKJ4*01 | 9 | CQQSYTTPLTF |
| TT-Ab5 | IGHV1-69*06 | IGHJ4*01 | IGHD5-12*01 | 13 | CATRESGYDGQFDYW | IGLV1-47*01 | IGLJ2*01 | 10 | CATWDDSLGVVF |
| TT-Ab6 | IGHV3-11*03 | IGHJ4*03 | IGHD6-19*01 | 15 | CARVGPWPVMKGAFDSW | IGKV3-20*01 | IGKJ3*01 | 10 | CQQYGSSPLFAF |
| TT-Ab7 | IGHV3-23*04 | IGHJ4*02 | IGHD6-13*01 | 13 | CARSVQQYLGLLGYW | IGLV4-69*01 | IGLJ3*02 | 9 | CQTWGAGMGMF |
| TT-Ab8 | IGHV3-48*03 | IGHJ4*02 | IGHD3-10*01 | 14 | CARDPSPGRLGFFDYW | IGKV3-15*01 | IGKJ1*01 | 9 | CQQYNNWPRTF |
| TT-Ab9 | IGHV3-7*03 | IGHJ4*02 | IGHD3-10*01 | 16 | CARGMTAWSLGGYQYDYW | IGKV1-6*01 | IGKJ1*01 | 9 | CLQNYNYPRTF |
| TT-Ab10 | IGHV3-7*03 | IGHJ4*02 | IGHD3-10*01 | 15 | CAREYGSYWRTYYFDDW | IGKV3-11*01 | IGKJ4*01 | 8 | CQQGSNWLTF |
| TT-Ab11 | IGHV4-31*03 | IGHJ4*02 | IGHD3-3*01 | 15 | CARVRGTGYYPGTLDLW | IGKV3-20*01 | IGKJ4*01 | 9 | CQQFGSSPLTF |
| TT-Ab12 | IGHV4-59*01 | IGHJ5*02 | IGHD3-22*01 | 19 | CARARYYYDSTGYFYGWLDPW | IGKV1-5*03 | IGKJ2*04 | 9 | CQQYKSYSCSF |
| TT-Ab13 | IGHV5-51*01 | IGHJ4*02 | IGHD3-22*01 | 16 | CARQGDPSSYLTSGFEFW | IGKV1-5*03 | IGKJ1*01 | 9 | CHQYNSYSQTF |

Table S2. **$K_{on}$, $K_{off}$, and $K_d$ of selected TT-specific antibodies binding to immobilized antigen were determined by BLI**

| Antibody | $K_d$ | $K_{on}$ | $K_{off}$ |
|---|---|---|---|
| | *M* | *1/M* | *1/s* |
| TT-Ab3 | $1.61 \times 10^{-9}$ | $8.05 \times 10^{4}$ | $1.30 \times 10^{-4}$ |
| TT-Ab4 | $1.37 \times 10^{-11}$ | $1.66 \times 10^{5}$ | $2.27 \times 10^{-6}$ |
| TT-Ab7 | $1.85 \times 10^{-11}$ | $1.87 \times 10^{5}$ | $3.46 \times 10^{-6}$ |
| TT-Ab9 | $6.64 \times 10^{-9}$ | $1.39 \times 10^{5}$ | $9.19 \times 10^{-4}$ |
| TT-Ab10 | $1.59 \times 10^{-9}$ | $1.53 \times 10^{5}$ | $2.43 \times 10^{-4}$ |
| TT-Ab12 | $1.95 \times 10^{-9}$ | $1.57 \times 10^{5}$ | $3.07 \times 10^{-4}$ |

Table S3. **Characteristics of the unique antigen-specific antibody sequences isolated from single plasma cells obtained after stimulation with particulate H1-CpG**

| Ab | VH name | JH name | DH name | CDR3 length | CDR3 sequence | VL name | JL name | CDR3 length | CDR3 sequence |
|---|---|---|---|---|---|---|---|---|---|
| | | | | *aa* | | | | *aa* | |
| H1-Ab1 | IGHV1-18*01 | IGHJ4*02 | IGHD3-9*01 | 15 | CARDRAHTLTGYHFDCW | IGKV2-30*01 | IGKJ2*01 | 10 | CMQGTHWPPYTF |
| H1-Ab2 | IGHV1-18*01 | IGHJ4*02 | IGHD3-9*01 | 15 | CARDRAHILTGYHFDYW | IGKV2-30*01 | IGKJ2*01 | 10 | CMQGTHWPPYTF |
| H1-Ab3 | IGHV1-18*01 | IGHJ4*02 | IGHD3-9*01 | 15 | CARDRAHILTGYHFDYW | IGKV3-20*01 | IGKJ5*01 | 10 | CQQYGSSHVITF |
| H1-Ab4 | IGHV1-18*01 | IGHJ5*01 | IGHD4-11*01 | 10 | CARDVHYRFDSW | IGLV2-8*01 | IGLJ3*02 | 9 | CSSYAGGSRVF |
| H1-Ab5 | IGHV1-18*01 | IGHJ4*02 | IGHD3-10*01 | 17 | CARDVPHPLDNSGSSHDSW | IGKV2-30*01 | IGKJ2*02 | 9 | CMQATYWPRTF |
| H1-Ab6 | IGHV1-18*04 | IGHJ3*02 | IGHD2-2*01 | 15 | CARDRPHILAPSAFDIW | IGKV2-30*01 | IGKJ2*01 | 9 | CMQGTHWPYTF |
| H1-Ab7 | IGHV1-18*04 | IGHJ4*02 | IGHD5-18*01 | 14 | CARGTWIQHLYYFDYW | IGLV3-21*01 | IGLJ2*01 | 11 | CQVWDNTHDHVVF |
| H1-Ab8 | IGHV1-2*02 | IGHJ4*02 | IGHD3-9*01 | 16 | CARANRGGYFAWAFFDFW | IGKV3-20*01 | IGKJ4*01 | 9 | CQQYGSSPLTF |
| H1-Ab9 | IGHV1-2*02 | IGHJ3*01 | IGHD5-18*01 | 12 | CARIQLRSDGFDLW | IGLV2-23*02 | IGLJ3*02 | 10 | CCSYAGSSFWVF |
| H1-Ab10 | IGHV1-69*01 | IGHJ6*02 | IGHD3-9*01 | 27 | CARAGTPSPRLVLQYFDWLFPYYYGMDVW | IGKV2D-29*01 | IGKJ4*01 | 9 | CMQSLQLPITF |
| H1-Ab11 | IGHV1-69*06 | IGHJ6*02 | IGHD1-1*01 | 18 | CAKGGPTTGTPYYVFGLDVW | IGKV2-24*01 | IGKJ2*01 | 8 | CMQATQFQTF |
| H1-Ab12 | IGHV1-69*09 | IGHJ4*02 | IGHD3-22*01 | 15 | CATYQGGGSGYFPLDSW | IGKV3-20*01 | IGKJ2*01 | 9 | CHQYGGFMYTF |
| H1-Ab13 | IGHV1-69*09 | IGHJ4*02 | IGHD1-26*01 | 16 | CTRTPHVSTGSPGDIDNW | IGKV3-11*01 | IGKJ1*01 | 9 | CQQRGNWLWTF |
| H1-Ab14 | IGHV1-69*09 | IGHJ5*02 | IGHD3-22*01 | 15 | CARGNFDDRGYYQFDPW | IGKV2-28*01 | IGKJ1*01 | 9 | CMQGLQTPWTF |
| H1-Ab15 | IGHV1-69*09 | IGHJ4*02 | IGHD3-22*01 | 17 | CATGPSYYYDANGSPTDYW | IGKV3-15*01 | IGKJ1*01 | 9 | CQQYNNWPWTF |
| H1-Ab16 | IGHV1-69*09 | IGHJ4*02 | IGHD3-10*01 | 13 | CAGGTSRWFGDVEYW | IGKV2-30*01 | IGKJ1*01 | 10 | CMQGSHWPPSF |
| H1-Ab17 | IGHV1-69*09 | IGHJ4*02 | IGHD1-26*01 | 11 | CAKGDTVGPTPNW | IGKV3-20*01 | IGKJ2*03 | 9 | CQQYGTFLYSF |
| H1-Ab18 | IGHV3-15*01 | IGHJ4*02 | IGHD2-8*01 | 14 | CATDHSSHYYDRPDYW | IGLV4-69*01 | IGLJ3*02 | 9 | CQTWDTGIHVF |
| H1-Ab19 | IGHV3-21*01 | IGHJ4*02 | IGHD4-23*01 | 17 | CATGVTSSYGGYSGYFDSW | IGKV1-33*01 | IGKJ4*01 | 9 | CQQYDNPFLTF |
| H1-Ab20 | IGHV3-21*01 | IGHJ4*02 | IGHD2-15*01 | 17 | CATLAGYCIGGTCDDFDYW | IGLV3-25*03 | IGLJ2*01 | 8 | CQSADSSVVF |
| H1-Ab21 | IGHV3-21*01 | IGHJ4*02 | IGHD3-10*01 | 15 | CARFGASGSLGGGFDYW | IGKV3-15*01 | IGKJ2*02 | 9 | CQQYHDWPRTF |
| H1-Ab22 | IGHV3-21*01 | IGHJ4*02 | IGHD3-10*01 | 15 | CARFGASGSLGGGFDYW | IGKV3-15*01 | IGKJ2*02 | 9 | CQQYHDWPRTF |
| H1-Ab23 | IGHV3-21*04 | IGHJ4*02 | IGHD4-23*01 | 17 | CATGVTSSYGGHPGYFDSW | IGKV1-33*01 | IGKJ4*01 | 9 | CQQYDDPFLTF |
| H1-Ab24 | IGHV3-23*01 | IGHJ6*01 | IGHD3-3*01 | 21 | CAKDTGHKIFGVVRPYYHGMDVW | IGKV3-20*01 | IGKJ2*01 | 10 | CQQYGSSPMYTF |
| H1-Ab25 | IGHV3-23*01 | IGHJ4*02 | IGHD3-10*01 | 16 | CAKLPPLGSGNYGAIDYW | IGLV7-43*01 | IGLJ3*02 | 10 | CLLYFGGVNWVF |
| H1-Ab26 | IGHV3-23*01 | IGHJ6*02 | IGHD3-3*01 | 21 | CAKDTGHTIFGVVRPYYNGMDVW | IGKV2-30*01 | IGKJ2*01 | 8 | CMQGTHPYTF |
| H1-Ab27 | IGHV3-23*01 | IGHJ6*02 | IGHD3-3*01 | 21 | CAKDTGHTIFGVVRPYYNGMDVW | IGKV3-20*01 | IGKJ2*01 | 10 | CQQYGGSPMYTF |
| H1-Ab28 | IGHV3-23*01 | IGHJ4*02 | IGHD3-3*01 | 16 | CAKDLGVIIPSGGKFDSW | IGKV3-15*01 | IGKJ1*01 | 10 | CQQYNNWPPITF |
| H1-Ab29 | IGHV3-23*04 | IGHJ6*02 | IGHD3-3*01 | 21 | CAKDTGHKIFGVVRPYYHGMDVW | IGKV3-20*01 | IGKJ2*01 | 10 | CQQYGISPMYTF |
| H1-Ab30 | IGHV3-23*04 | IGHJ4*02 | IGHD6-19*01 | 16 | CAKALATHIVMAGTLDFW | IGKV3-15*01 | IGKJ4*01 | 10 | CQQYNNWPPLTF |
| H1-Ab31 | IGHV3-23*04 | IGHJ4*02 | IGHD3-3*01 | 20 | CAKDRGRSIFGLVTQDYHFDYW | IGKV1-39*01 | IGKJ4*01 | 9 | CQQSFRSPLTF |
| H1-Ab32 | IGHV3-23*04 | IGHJ4*02 | IGHD3-3*01 | 20 | CARVVGRETFGLVIPIYYFDYW | IGKV3-11*01 | IGKJ4*01 | 10 | CQQCSNWPPLTF |
| H1-Ab33 | IGHV3-23*04 | IGHJ4*02 | IGHD3-3*01 | 20 | CAKDQGNAIFGLVIPSYYFDYW | IGKV3-20*01 | IGKJ4*01 | 10 | CQQYGSSPQLTF |
| H1-Ab34 | IGHV3-23*04 | IGHJ4*02 | IGHD3-3*01 | 18 | CAKEVSWITIFGVITPFDSW | IGKV3-15*01 | IGKJ4*01 | 9 | CQQCNNWPLTF |
| H1-Ab35 | IGHV3-23*04 | IGHJ1*01 | IGHD3-3*01 | 14 | CAQDAITFFGVIITSW | IGKV3-15*01 | IGKJ1*01 | 9 | CQQYNEWPPTF |
| H1-Ab36 | IGHV3-23*04 | IGHJ6*02 | IGHD3-3*01 | 20 | CAKGDEFWSGYSPSYSYAMDVW | IGLV2-14*01 | IGLJ2*01 | 10 | CSSYTGSSTLVVF |
| H1-Ab37 | IGHV3-23*04 | IGHJ4*02 | IGHD3-3*01 | 20 | CAKDQGNAIFGLVIPSYYFDYW | IGKV3-20*01 | IGKJ4*01 | 10 | CQQYGSSPQLTF |
| H1-Ab38 | IGHV3-30-3*01 | IGHJ5*01 | IGHD5-18*01 | 9 | CARRYDGFDYW | IGLV2-23*02 | IGLJ2*01 | 10 | CCSYAGSYTYVF |
| H1-Ab39 | IGHV3-30*01 | IGHJ2*01 | IGHD3-22*01 | 16 | CARGNCRPRGYYIPQDLW | IGKV1-5*03 | IGKJ1*01 | 9 | CHQYDGFPWTF |
| H1-Ab40 | IGHV3-30*01 | IGHJ6*02 | IGHD2-2*01 | 24 | CARDVVNCSGTTCRNMDYYYYGMDVW | IGLV3-1*01 | IGLJ2*01 | 11 | CQAWDSSTDHVEF |
| H1-Ab41 | IGHV3-33*01 | IGHJ4*02 | IGHD3-22*01 | 20 | CARGFLLPYYGSSGYEYFFDFW | IGKV4-1*01 | IGKJ4*01 | 8 | CQQYYSTPSF |
| H1-Ab42 | IGHV3-48*02 | IGHJ6*02 | IGHD4-17*01 | 18 | CARAGGGYGDYFYFYGMDVW | IGLV3-21*01 | IGLJ2*01 | 11 | CQVWDSSSDHVIF |
| H1-Ab43 | IGHV3-48*03 | IGHJ4*02 | IGHD6-19*01 | 13 | CVSPIPVAGTMEIYW | IGKV3-15*01 | IGKJ4*01 | 10 | CQQYNNWPPLTF |
| H1-Ab44 | IGHV3-64*04 | IGHJ5*02 | IGHD1-26*01 | 16 | CSRGGIVRYSGSYGPDVW | IGKV2-28*01 | IGKJ1*01 | 9 | CMQALQMPWTF |
| H1-Ab45 | IGHV3-66*01 | IGHJ3*02 | IGHD5-24*01 | 17 | CARGGDAYNSGPTGAFDIW | IGLV3-21*01 | IGLJ3*02 | 11 | CQVWHSISDHWLF |
| H1-Ab46 | IGHV3-7*03 | IGHJ4*02 | IGHD2-21*02 | 13 | CARYCGGDCFGFDYW | IGKV3-15*01 | IGKJ1*01 | 10 | CQQYNDWPSWTF |
| H1-Ab47 | IGHV3-9*01 | IGHJ6*02 | IGHD1-26*01 | 12 | CVKDVGGYYAMDVW | IGLV1-51*01 | IGLJ1*01 | 11 | CGTWDSNLKAYIF |
| H1-Ab48 | IGHV3-9*01 | IGHJ4*02 | IGHD6-19*01 | 13 | CAKADSGWYGYFDSW | ND | | | |
| H1-Ab49 | IGHV3-9*01 | IGHJ4*02 | IGHD6-19*01 | 14 | CVKDRGLGGWYFFESW | IGLV1-51*01 | IGLJ1*01 | 11 | CGTWDSNLKAYIF |
| H1-Ab50 | IGHV4-31*03 | IGHJ3*02 | IGHD5-24*01 | 14 | CATDSKVGYVEAPDIW | IGLV1-44*01 | IGLJ2*01 | 11 | CAAWDDNLNGVLF |
| H1-Ab51 | IGHV4-39*01 | IGHJ3*02 | IGHD2-21*02 | 16 | CARSYCGGGCNFDAFDIW | IGLV2-14*01 | IGLJ3*02 | 10 | CSSYTSNITLVF |
| H1-Ab52 | IGHV4-39*03 | IGHJ3*02 | IGHD4-23*01 | 11 | CGTSVNLDAVHIW | IGLV2-14*01 | IGLJ2*01 | 11 | CSSYTDINTHVIF |
| H1-Ab53 | IGHV4-39*03 | IGHJ5*02 | IGHD3-3*01 | 20 | CARMSYMDYDFSTGYWNWFDPW | IGLV2-23*01 | IGLJ3*02 | 10 | CCSYAGSSTWVF |
| H1-Ab54 | IGHV4-39*03 | IGHJ5*02 | IGHD3-3*01 | 20 | CARLSYMDYDFSTGYWNWFDPW | IGLV2-23*01 | IGLJ3*02 | 10 | CCSYAGSSTWVF |
| H1-Ab55 | IGHV4-59*01 | IGHJ3*01 | IGHD1-1*01 | 15 | CARDQTGIRKSNAFDLW | IGKV1-39*01 | IGKJ1*01 | 9 | CQQSYDTPQTF |
| H1-Ab56 | IGHV4-59*01 | IGHJ3*02 | IGHD6-6*01 | 15 | CARDQAGIRRSNAFDIW | IGKV1-39*01 | IGKJ1*01 | 9 | CQQSYDIPQTF |
| H1-Ab57 | IGHV4-61*02 | IGHJ4*02 | IGHD3-22*01 | 13 | CARLSISSGYYYDYW | IGLV6-57*01 | IGLJ2*01 | 9 | CQSYDTFNHVF |
| H1-Ab58 | IGHV4-61*02 | IGHJ4*02 | IGHD6-19*01 | 14 | CAREDSSGWEYYFDYW | IGKV3-20*01 | IGKJ2*03 | 9 | CQQYGTFLYSF |
| H1-Ab59 | IGHV4-61*02 | IGHJ4*02 | IGHD3-16*02 | 19 | CAREDDFVWGSYRRFAYFDYW | IGKV1-33*01 | IGKJ2*01 | 10 | CQQYDNLPMYTF |
| H1-Ab60 | IGHV4-61*07 | IGHJ5*02 | IGHD4-17*01 | 15 | CARDRGELRGYNWLDPW | ND | | | |

Table S3. **Characteristics of the unique antigen-specific antibody sequences isolated from single plasma cells obtained after stimulation with particulate H1-CpG** *(Continued)*

| Ab | VH name | JH name | DH name | CDR3 length | CDR3 sequence | VL name | JL name | CDR3 length | CDR3 sequence |
|---|---|---|---|---|---|---|---|---|---|
| H1-Ab61 | IGHV5-51*01 | IGHJ6*02 | IGHD3-16*02 | 16 | CARHPGDKSFYYYGLDVW | IGKV1-5*03 | IGKJ5*01 | 9 | CQQYNSDSITF |
| H1-Ab62 | IGHV5-51*01 | IGHJ4*02 | IGHD6-19*01 | 13 | CARQYTSGWYILDYW | IGLV1-44*01 | IGLJ2*01 | 11 | CAAWDDSLSGVLF |

Table S4. **$K_{on}$, $K_{off}$, and $K_d$ of selected H1-specific antibodies binding to immobilized antigen were determined by SPR measurements**

| Antibody | $K_d$ | $K_{on}$ | $K_{off}$ |
|---|---|---|---|
| | *M* | *1/M* | *1/s* |
| H1-Ab10 | $5.6 \times 10^{-4}$ | 9 | |
| H1-Ab19 | $1 \times 10^{-12}$ | $4.77 \times 10^{4}$ | |
| H1-Ab20 | $6.88 \times 10^{-4}$ | $2.00 \times 10^{5}$ | |
| H1-Ab24 | $8.36 \times 10^{-4}$ | $2.37 \times 10^{5}$ | |
| H1-Ab29 | $7.36 \times 10^{-4}$ | $2.98 \times 10^{5}$ | |
| H1-Ab30 | $6.6 \times 10^{-11}$ | $1.42 \times 10^{5}$ | |
| H1-Ab32 | $5.6 \times 10^{-10}$ | $2.17 \times 10^{3}$ | |
| H1-Ab41 | $8.19 \times 10^{-11}$ | $2.72 \times 10^{5}$ | |
| H1-Ab43 | $1.55 \times 10^{-10}$ | $4.82 \times 10^{4}$ | |
| H1-Ab44 | $2.11 \times 10^{-4}$ | $2.24 \times 10^{4}$ | |
| H1-Ab54 | $2.97 \times 10^{-7}$ | $1.11 \times 10^{3}$ | $3.30 \times 10^{-4}$ |
| H1-Ab55 | $4.67 \times 10^{-10}$ | | |
| H1-Ab56 | $3.72 \times 10^{-10}$ | $2.78 \times 10^{4}$ | |
| H1-Ab59 | $1.4 \times 10^{-8}$ | | |

Table S5. **Characteristics of the unique antigen-specific antibody sequences isolated from single plasma cells obtained after stimulation with particulate H5-CpG**

| Ab | VH name | JH name | DH name | CDR3 length | CDR3 sequence | VL name | JL name | CDR3 length | CDR3 sequence |
|---|---|---|---|---|---|---|---|---|---|
| | | | | *aa* | | | | *aa* | |
| H5-Ab1 | IGHV1-18*01 | IGHJ5*01 | IGHD6-25*01 | 18 | CAVDSGGYFSSPRYRWIDPW | IGLV1-51*01 | IGLJ1*01 | 11 | CGTWDSNLKAYIF |
| H5-Ab2 | IGHV1-69*01 | IGHJ4*02 | IGHD3-10*01 | 17 | CASAHGYHNYGSRGYFVSW | IGKV3-20*01 | IGKJ2*01 | 9 | CQQYGSSPHTF |
| H5-Ab3 | IGHV3-21*01 | IGHJ5*02 | IGHD2-15*01 | 14 | CARDRGESSYYWFDPW | IGLV2-23*02 | IGLJ3*02 | 10 | CSSYAGTTIWVF |
| H5-Ab4 | IGHV3-30*03 | IGHJ4*02 | IGHD3-9*01 | 21 | CAKDFQLRLLRYFDWLSSPFDHW | IGKV3-20*01 | IGKJ1*01 | 9 | CQQYASSPQTF |
| H5-Ab5 | IGHV3-33*01 | IGHJ4*02 | IGHD3-9*01 | 23 | CARSGFRGIALRYFDWQRTDYFDGW | IGLV2-23*01 | IGLJ3*02 | 10 | CCSYAGSSTWVF |
| H5-Ab6 | IGHV5-10-1*01 | IGHJ1*01 | IGHD3-9*01 | 21 | CARQAGTLLRYFESRTKYPFQDW | IGKV3-20*01 | IGKJ2*01 | 10 | CQQYGDSPAYTF |

Table S6. **Characteristics of the unique antigen-specific antibody sequences isolated from single plasma cells obtained after stimulation with particulate H7-CpG**

| Ab | VH name | JH name | DH name | CDR3 length | CDR3 sequence | VL name | JL name | CDR3 length | CDR3 sequence |
|---|---|---|---|---|---|---|---|---|---|
| | | | | *aa* | | | | *aa* | |
| H7-Ab1 | IGHV1-3*01 | IGHJ5*02 | IGHD3-10*01 | 13 | CAHGSGSYLNWFDPW | IGLV2-14*01 | IGLJ2*01 | 11 | CTSYTSSSTLVAF |
| H7-Ab2 | IGHV1-3*01 | IGHJ1*01 | IGHD5-18*01 | 19 | CARDRRWWGEGDTAVEYFQYW | IGKV3-15*01 | IGKJ1*0 | 9 | CQQYNNWPPTL |
| H7-Ab3 | IGHV1-69*01 | IGHJ4*03 | IGHD3-3*01 | 15 | CSRVADDYDFWSPNKFW | IGKV1-5*03 | IGKJ1*01 | 8 | CQQYNSNSHF |
| H7-Ab4 | IGHV1-69*01 | IGHJ4*02 | IGHD3-16*01 | 12 | CARIHRAGLEANYW | IGKV1-5*01 | IGKJ1*01 | 9 | CQQYNTYSWTF |
| H7-Ab5 | IGHV1-69*01 | IGHJ6*02 | IGHD6-25*01 | 19 | CARAPVVGSRNVYYYHGLDVW | IGLV3-25*03 | IGLJ3*02 | 10 | CQSADSSDPCLF |
| H7-Ab6 | IGHV3-21*01 | IGHJ2*02 | IGHD1-7*01 | 16 | CARGAKVELALGWYFDLW | IGLV1-51*01 | IGLJ3*01 | 11 | CGTWDNRLSAAVF |
| H7-Ab7 | IGHV3-23*01 | IGHJ5*02 | IGHD1-26*01 | 14 | CAKDASWDVRGWFDPW | IGKV4-1*01 | IGKJ2*01 | 9 | CQQYYSTPYTF |
| H7-Ab8 | IGHV3-33*01 | IGHJ4*02 | IGHD3-3*01 | 13 | CAREVIYSGYFFDHW | IGKV3-15*01 | IGKJ1*01 | 10 | CQQYTYWPPWTF |
| H7-Ab9 | IGHV3-33*01 | IGHJ6*02 | IGHD3-10*01 | 12 | CARDPVGRYGMDVW | IGKV1-6*01 | IGKJ1*01 | 9 | CLQNYNFPWTF |
| H7-Ab10 | IGHV3-7*01 | IGHJ2*01 | IGHD2-21*01 | 10 | CATSSGWRFEVW | IGLV2-8*01 | IGLJ2*01 | 10 | CSSYAGTKHWIF |
| H7-Ab11 | IGHV4-31*03 | IGHJ4*02 | IGHD1-26*01 | 23 | CARTQRSTVAHIVGPVQGRFHFDYW | IGKV3-20*01 | IGKJ3*01 | 11 | CQQYGRSSGFVF |
| H7-Ab12 | IGHV4-31*01 | IGHJ4*02 | IGHD2-21*01 | 8 | CARVRGVYFW | IGKV3-20*01 | IGKJ1*01 | 11 | CQQYGSSLPPWTF |
| H7-Ab13 | IGHV4-31*02 | IGHJ5*02 | IGHD3-3*01 | 21 | CARNSAYCTDTRCYNGGGWFHPW | IGKV3-20*01 | IGKJ5*01 | 9 | CQQYGSSPITF |
| H7-Ab14 | IGHV4-39*01 | IGHJ3*02 | IGHD5-24*01 | 14 | CAVIQLSTPPVPFEIW | IGKV3-20*01 | IGKJ2*01 | 9 | CHQYDSSWYTF |

Table S7. **Characteristics of the unique antigen-specific antibody sequences isolated from single plasma cells obtained after stimulation with particulate gp120-CpG**

| Ab | VH name | JH name | DH name | CDR3 length | CDR3 sequence | VH mutations | VL name | JL name |
|---|---|---|---|---|---|---|---|---|
| | | | | *aa* | | *n* | | |
| gp120-Ab1 | IGHV3-11*06 | IGHJ3*02 | IGHD5-18*01 | 22 | CARDRTPRGPRRVETAMVDAFDIW | 11 | IGKV1-12*01 | IGKJ4*01 |
| gp120-Ab2 | IGHV3-23*04 | IGHJ4*02 | IGHD2-15*01 | 18 | CAKRPGYCSGGSCFVYFDYW | 12 | IGLV1-51*01 | IGLJ2*01 |
| gp120-Ab3 | IGHV3-23*04 | IGHJ4*02 | IGHD2-21*01 | 17 | CAKDLADFRGWRLRIYDYW | 34 | IGLV2-11*01 | IGLJ3*02 |
| gp120-Ab4 | IGHV3-23*04 | IGHJ4*02 | IGHD3-16*02 | 24 | CARVRSSGHVKITFGGIIVYRYFDNW | 19 | IGKV1-33*01 | IGKJ1*01 |
| gp120-Ab5 | IGHV3-23*04 | IGHJ5*02 | IGHD6-19*01 | 21 | CAKDSGSYGRQWLARRQGWFDPW | 27 | IGKV1-5*03 | IGKJ3*01 |
| gp120-Ab6 | IGHV3-30*03 | IGHJ1*01 | IGHD3-10*01 | 11 | CAKDNGAILPHDW | 5 | IGLV2-23*02 | IGLJ2*01 |
| gp120-Ab7 | IGHV3-30*03 | IGHJ4*02 | IGHD3-3*01 | 16 | YCAKDSPHASGNYPPDSW | 14 | IGKV1-13*02 | IGKJ1*01 |
| gp120-Ab8 | IGHV3-30*03 | IGHJ5*02 | IGHD1-26*01 | 15 | CAKSLVGATLLGPFGPW | 14 | IGLV3-21*02 | IGLJ1*01 |
| gp120-Ab9 | IGHV3-30*03 | IGHJ4*02 | IGHD1-26*01 | 15 | CARSLVGASSRGPYGYW | 7 | IGLV3-21*02 | IGLJ1*01 |
| gp120-Ab10 | IGHV3-33*01 | IGHJ6*02 | IGHD3-10*01 | 14 | CVRGTVPHYYYGMTLW | 24 | IGLV5-45*01 | IGLJ3*02 |
| gp120-Ab11 | IGHV3-64D*06 | IGHJ4*02 | IGHD3-9*01 | 15 | CVKDIKKTGDSKRFDYW | 0 | IGLV1-51*01 | IGLJ3*02 |
| gp120-Ab12 | IGHV3-64D*06 | IGHJ5*02 | IGHD3-9*01 | 15 | CVKDIKKTGDYKRFDHW | 3 | IGLV1-51*01 | IGLJ3*02 |
| gp120-Ab13 | IGHV3-64D*06 | IGHJ4*02 | IGHD3-9*01 | 15 | CVKDIKKTGDSKRFDYW | 0 | IGLV1-51*01 | IGLJ3*02 |
| gp120-Ab14 | IGHV3-7*01 | IGHJ4*02 | IGHD6-19*01 | 11 | CVRGVSAVAGPYW | 3 | IGLV1-51*01 | IGLJ6*01 |
| gp120-Ab15 | IGHV4-34*01 | IGHJ4*02 | IGHD3-3*01 | 18 | CARNKAGRFRQQLTTPLDYW | 2 | IGKV3-20*01 | IGKJ1*01 |
| gp120-Ab16 | IGHV5-51*01 | IGHJ4*02 | IGHD1-26*01 | 12 | CARCTGEGNYFQLW | 29 | IGKV1-33*01 | IGKJ2*01 |
| gp120-Ab17 | IGHV5-51*01 | IGHJ4*02 | IGHD6-13*01 | 13 | CASTAYSSTWYIYYW | 6 | IGKV3-20*01 | IGKJ4*01 |
| gp120-Ab18 | IGHV5-51*01 | IGHJ5*02 | IGHD2-2*01 | 17 | CARHMGRYCSSNTCPFDPW | 13 | IGKV1-6*01 | IGKJ2*01 |

**Table S8, included as an Excel file, shows sequences of all antibodies isolated in this study.**
